# Pharmacological Characterization of 5-Substituted 1-[(2,3-dihydro-1-benzofuran-2-yl)methyl]piperazines: Novel Antagonists for the Histamine H_3_ and H_4_ Receptors with Anti-inflammatory Potential

**DOI:** 10.3389/fphar.2017.00825

**Published:** 2017-11-14

**Authors:** Michelle F. Corrêa, Álefe J. R. Barbosa, Larissa B. Teixeira, Diego A. Duarte, Sarah C. Simões, Lucas T. Parreiras-e-Silva, Aleksandro M. Balbino, Richardt G. Landgraf, Michel Bouvier, Claudio M. Costa-Neto, João P. S. Fernandes

**Affiliations:** ^1^Departamento de Ciências Farmacêuticas, Universidade Federal de São Paulo, Diadema, Brazil; ^2^Departamento de Bioquímica e Imunologia, Faculdade de Medicina de Ribeirão Preto, Universidade de São Paulo, Ribeirão Preto, Brazil; ^3^Department of Biochemistry and Molecular Medicine, Institute for Research in Immunology and Cancer, University of Montréal, Montréal, QC, Canada

**Keywords:** H_3_R antagonists, H_4_R antagonists, histamine receptors, dihydrobenzofuran, SAR, anti-inflammatory activity, asthma

## Abstract

The histamine receptors (HRs) are traditional G protein-coupled receptors of extensive therapeutic interest. Recently, H_3_R and H_4_R subtypes have been targeted in drug discovery projects for inflammation, asthma, pain, cancer, Parkinson’s, and Alzheimer’s diseases, which includes searches for dual acting H_3_R/H_4_R ligands. In the present work, nine 1-[(2,3-dihydro-1-benzofuran-2-yl)methyl]piperazine (LINS01 series) molecules were synthesized and evaluated as H_3_R and H_4_R ligands. Our data show that the *N*-allyl-substituted compound LINS01004 bears the highest affinity for H_3_R (p*K*_i_ 6.40), while the chlorinated compound LINS01007 has moderate affinity for H_4_R (p*K*_i_ 6.06). In addition, BRET assays to assess the functional activity of G_i_1 coupling indicate that all compounds have no intrinsic activity and act as antagonists of these receptors. Drug-likeness assessment indicated these molecules are promising leads for further improvements. *In vivo* evaluation of compounds LINS01005 and LINS01007 in a mouse model of asthma showed a better anti-inflammatory activity of LINS01007 (3 g/kg) than the previously tested compound LINS01005. This is the first report with functional data of these compounds in HRs, and our results also show the potential of their applications as anti-inflammatory.

## Introduction

Histamine is one of the most important chemical transmitters involved in several biological processes. It has been widely studied since its discovery^1-3^ due to its role in inflammatory and allergic reactions, but it is also involved in the regulation of gastric acid secretion, sleep, mood, and food intake (Passani and Blandina, 2011). These processes are triggered by the interaction of histamine with its receptors (HRs), named H_1_R, H_2_R, H_3_R, and H_4_R. HRs are members of the G protein-coupled receptor (GPCR) class A family, which are also known as 7 transmembrane (7TM) receptors (Seifert et al., 2011; Corrêa and Fernandes, 2015).

**FIGURE 1:**
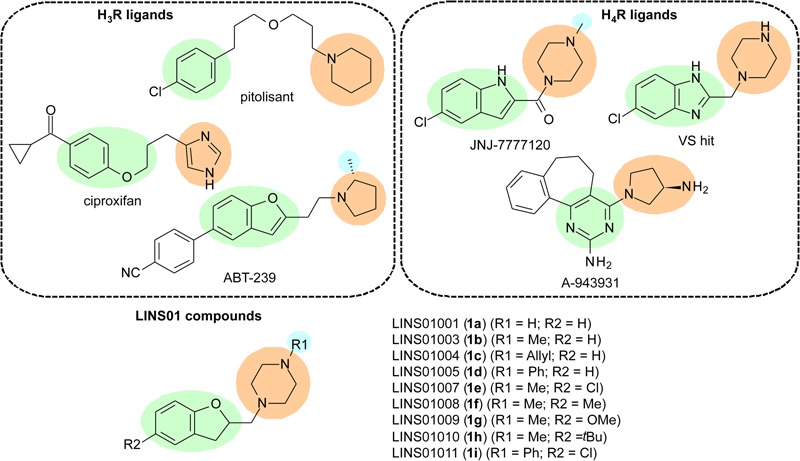
Characteristics considered in the design of LINS compounds **1a**–**i**; green – aromatic core; orange – polar group; blue – additional lipophilic group.

The H_3_R is mainly found in CNS, and is involved in the inhibition of histamine release, as well as in the inhibition of other neurotransmitters, such as norepinephrine, acetylcholine, and serotonin (Leurs et al., 2005). Therefore, H_3_R modulates neurotransmitters release by acting as a presynaptic receptor (auto- and hetero-receptor). It has been previously reported that signal transduction by H_3_R occurs mainly by activation of G_i/o_ proteins, leading to decrease of intracellular cAMP concentration and decrease of Ca^2+^ influx in neurons (Tiligada et al., 2009). As a result of its actions, H_3_R could be involved in several neurological disorders, including cognitive, convulsive, and sleep–wake disorders, as well as in obesity (Łażewska et al., 2014). Therefore, H_3_R ligands could be promising drugs to treat these conditions. H_3_R ligands have been widely explored. Recently, the novel H_3_R antagonist pitolisant has been approved for treatment of narcolepsy, highlighting the importance of this receptor as a new target for treatment of certain CNS disorders (Sayed, 2016). Ciproxifan and ABT-239 (**Figure 1**) are other examples of such well-studied compounds.

In the early 2000s, several research groups described a new isoform of HR, expressed in immune cells (eosinophils, basophils, mast cells, NK cells, DCs, monocytes, and T cells), which is involved in the modulation of chemotaxis as well as other functions (Oda et al., 2000; Liu et al., 2001). This receptor was named H_4_R, is also coupled to G_i/o_ proteins (Corrêa and Fernandes, 2015), and bears a considerable sequence identity to H_3_R (∼31% total, 54% in TM domains) (Oda et al., 2000; Liu et al., 2001). Considering the physico-chemical properties of the different amino acids, this similarity in TM domains can reach 68% (de Esch et al., 2005). Since these cells are involved in immune and inflammatory processes, H_4_R is considered a promising target to future anti-inflammatory and modulatory immunological drugs. H_4_R ligands have been designed and evaluated as drug candidates (**Figure 1**), with the indolecarboxamides (JNJ-7777120) and 2-aminopyrimidines (A-943931) being the most explored chemotypes in this regard. Some molecules lacking the carbonyl group from the indolecarboxamides were also identified as H_4_R ligands in virtual screening experiments (Christopher et al., 2012).

Due to the high sequence identity between H_3_R and H_4_R, it is likely that several compounds that bind to one of them can present considerable affinity for the other. Hence, some pharmacophore templates to H_3_R ligands can also be applied to H_4_R ligands (Kottke et al., 2011). In fact, the search for selective ligands that are able to discriminate one of those receptors is frequently reported in the literature, and most medicinal chemists working with HR-ligand research have such selectivity parameter as one of the most important goals. However, dual-acting H_3_R/H_4_R ligands may also present therapeutic potential in determined pathological conditions, such as neuropathic pain (Smith et al., 2007), cancer (Medina et al., 2008), and have also been reported in Parkinson’s disease (Shan et al., 2015a,b). Such potential applications were already reported for imidazole-containing H_3_R/H_4_R ligands, such as imetit, immepip, clobenpropit, and thioperamide.

To the best of our knowledge, the functional activity of dihydrobenzofuran-containing molecules have not yet evaluated in the H_3_R and H_4_R until the present report. The affinities to these receptors were evaluated as well. Considering the chemical similarities observed for several H_3_R and H_4_R ligands, we have designed a set of 1-[(2,3-dihydro-1-benzofuran-2-yl)methyl]piperazines (LINS01 series – **1a**–**i**), evaluated their affinity and selectivity for those receptors, and characterized their functional activities. Drug-likeness was also assessed to define some structure-activity relationship (SAR) roles in this set of compounds, and an evaluation of the anti-inflammatory potential of a selected molecule was carried out in a mouse asthma model.

## Materials and Methods

Reagents and starting materials were obtained from commercial suppliers (Sigma–Aldrich Co., Saint Louis, MO, United States; LabSynt Co., Diadema, Brazil) and used without further purification. ^1^H and ^13^C NMR spectra were recorded in a Bruker Ultrashield 300 spectrometer, operating at 300 and 75 MHz, respectively, using CDCl_3_ as solvent with TMS as internal standard. Chemical shifts are reported in parts per million (ppm, δ units). Coupling constants (*J*) are reported in units of hertz (Hz), if applicable. The high resolution mass spectra (HRMS) were obtained through direct injection after electron-spray ionization in positive mode (ESI+) in a MicroTOF from Bruker Daltonics mass spectrometer. Gas chromatography coupled to mass spectrometer (GC-MS) analysis were done in a Shimadzu GC-2010 coupled to mass spectrometer GCMS-QP2010 plus, using helium as carrier gas in a silica capillary column. The low resolution mass spectra (LRMS) were obtained through electron impact ionization (70 eV). The ion-radical and its fragments are reported in mass/charge ratio (*m/z*). The purity (>95%) for the final compounds **1a–i** were determined and confirmed (H_2_O/MeOH 50%) by HPLC in a C-18 column coupled to UV detector (254 nm). Only compounds with >95% purity were considered to the biological assays. The yields for each step are summarized in **Table 1**. Spectral data and characterization for the intermediates can be found in Supplementary Information.

**Table 1:** Yields obtained for the synthesis of compounds **1a–i**.

Compounds	Yield (%)			
	Step a	Step b	Step c	Step d
**1a**	–	–	90	83
**1b**				85
**1c**				66
**1d**				50
**1e**	86	80	89	59
**1f**	87	79	88	50
**1g**	70	85	92	65
**1h**	84	73	89	69
**1i**	86	80	89	45

### Synthetic Procedure for Intermediates 2–5

**FIGURE 2:**
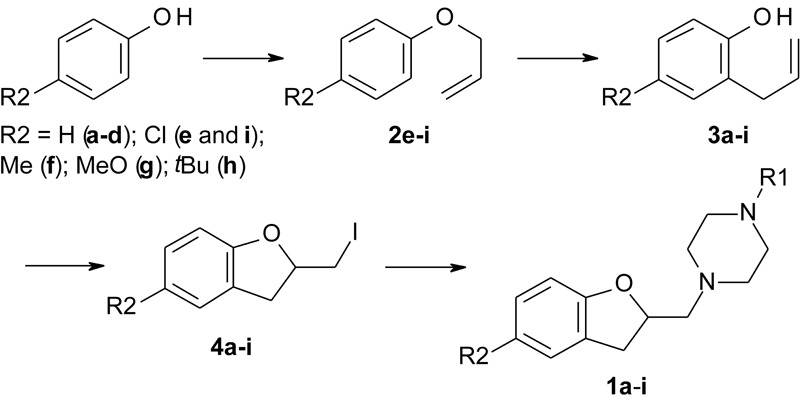
Reaction scheme for the synthesis of LINS01 compounds.

The intermediate compounds (**Figure 2** and Supplementary Figure S1) were synthesized according to previously reported procedures from our group (Corrêa et al., 2016, 2017). The experimental details can be found in the Supplementary Information.

### General Procedure for the Synthesis of Final Products (1a–i)

In a round-bottom flask, were added 2 mmol of corresponding 2-iodomethyl-2,3-dihydrobenzofuran (**4a–h**), 2.8 mmol (0.379 g) of K_2_CO_3_, and 8 mmol of corresponding 1-substituted piperazine in 15 mL of tetrahydrofuran. The mixture was reacted at 70°C for 18–24 h. The reactional mixture was filtered, the solvent removed under vacuum, and the residue was taken up in 1 M HCl solution (pH < 2), and washed with 2 × 10 mL of hexane. The aqueous phase was alkalinized (pH > 12) with 1 M NaOH solution, and extracted 3 × 10 mL of ethyl acetate. The organic layer was dried with anhydrous Na_2_SO_4_ and filtered. The solution was filtered over silica gel to remove any piperazine remaining, and the solvent removed under vacuum. If necessary, column chromatography in silica gel was used to purify the compounds, using dichloromethane:methanol (8:1) as eluent (**Figure 2**).

1-[(2,3-Dihydro-1-benzofuran-2-yl)methyl]piperazine (**1a**). Yellowish solid. mp 118–121°C. ^1^H NMR (CDCl_3_, 300 MHz): δ 2.45–2.70 (m, 8H), 2.79 (dd, 1H, *J* = 13.2, 7.7 Hz), 2.88–3.00 (m, 2H), 3.27 (dd, 1H, *J* = 15.7, 9.3 Hz), 4.89–5.04 (m, 1H), 6.77–6.88 (m, 2H), 7.08–7.20 (m, 2H). ^13^C NMR (CDCl_3_, 75 MHz): δ 34.3, 51.4, 53.9, 63.4, 80.8, 109.6, 120.3, 124.9, 126.5, 127.9, 159.6. LRMS (EI) *m/z* (rel %): 218 (15) [M^+^], 99 (100), 70 (26). HRMS (ESI+) for C_13_H_18_N_2_O [M+H]^+^: calcd 219.1493; found 219.1523.

1-[(2,3-dihydro-1-benzofuran-2-yl)methyl]-4-methylpiperazine (**1b**). Yellowish solid. mp 45–49°C. ^1^H NMR (CDCl_3_, 300 MHz): δ 2.30 (s, 3H), 2,49 (br.s, 4H), 2.57 (dd, 1H, *J* = 13.4, 4.4 Hz), 2.63 (br.s, 4H), 2.79 (dd, 1H, *J* = 13.4, 7.8 Hz), 2.94 (dd, 1H, *J* = 15.6, 7.8 Hz), 3.26 (dd, 1H, *J* = 15.6, 9.2 Hz), 4.95 (dq, 1H, *J* = 9.0, 3.9 Hz), 6.75–6.85 (m, 2H), 7.06–7.20 (m, 2H). ^13^C NMR (CDCl_3_, 75 MHz): δ 34.2, 46.0, 53.8, 55.0, 63.2, 80.8, 109.6, 120.3, 124.9, 126.4, 128.0, 159.5. LRMS (EI) *m/z* (rel %): 232 (18) [M^+^], 113 (95), 70 (100). HRMS (ESI+) for C_14_H_20_N_2_O [M+H]^+^: calcd 233.1649; found 233.1683.

1-allyl-4-[(2,3-dihydro-1-benzofuran-2-yl)methyl]piperazine (**1c**). Yellowish oil. ^1^H NMR (CDCl_3_, 300 MHz): δ 2.38–2.72 (m, 8H), 2.57 (dd, 1H, *J* = 13.3, 4.1 Hz), 2.79 (dd, 1H, *J* = 13.3, 8.1 Hz), 2.94 (dd, 1H, *J* = 15.6, 8.1 Hz), 3.01 (dq, 2H, *J* = 6.6, 1.4 Hz), 3.26 (dd, 1H, *J* = 15.5, 9.1 Hz), 4.95 (dq, 1H, *J* = 9.1, 4.1 Hz), 5.09–5.26 (m, 2H), 5.78–5.96 (m, 1H), 6.75–6.88 (m, 2H), 7.05–7.21 (m, 2H). ^13^C NMR (CDCl_3_, 75 MHz): δ 34.2, 53.0, 53.8, 61.8, 63.3, 80.8, 109.7, 118.0, 120.3, 124.9, 126.5, 128.0, 135.1, 159.5. LRMS (EI) *m/z* (rel %): 258 (23) [M^+^], 139 (100), 70 (27). HRMS (ESI+) for C_16_H_22_N_2_O [M+H]^+^: calcd 259.1806; found 259.1840.

1-[(2,3-dihydro-1-benzofuran-2-yl)methyl]-4-phenyl-piperazine (**1d**). Yellowish solid. mp 47–51°C. ^1^H NMR (CDCl_3_, 300 MHz): δ 2.64 (dd, 1H, *J* = 13.4, 4.2 Hz) 2.69–2.83 (m, 4H), 2.85 (dd, 1H, *J* = 13.2, 7.7 Hz), 3.05 (dd, 1H, *J* = 15.5, 7.7 Hz), 3.21–3.35 (m, 5H), 4.95–5.06 (m, 1H), 6.76–6.90 (m, 3H), 6.91–6.97 (m, 2H), 7.07–7.20 (m, 2H), 7.23–7.31 (m, 2H). ^13^C NMR (CDCl_3_, 75 MHz): δ 34.2, 49.1, 53.9, 63.2, 80.8, 109.7, 116.1, 119.7, 120.4, 124.9, 126.4, 128.1, 129.1, 151.4, 159.5. LRMS (EI) *m/z* (rel %): 294 (25) [M^+^], 175 (100), 70 (69). HRMS (ESI+) for C_19_H_22_N_2_O [M+H]^+^: calcd 295.1806; found 295.1846.

1-[(5-chloro-2,3-dihydro-1-benzofuran-2-yl)methyl]-4-methylpiperazine (**1e**). Yellow oil. ^1^H NMR (CDCl_3_, 300 MHz): δ 2.33 (s, 3H), 2.55 (br.s, 4H), 2.57 (dd, 1H, *J* = 13.4, 4.2 Hz), 2.65 (br.s, 4H), 2.78 (dd, 1H, *J* = 13.4, 7.7 Hz), 2.94 (dd, 1H, *J* = 15.9, 8.0 Hz), 3.24 (dd, 1H, *J* = 15.9, 9.1 Hz), 4.91–5.03 (m, 1H), 6.68 (d, 1H, *J* = 8.4 Hz), 7.04 (dd, 1H, *J* = 8.4, 2.3 Hz), 7.09–7.11 (m, 1H).^13^C NMR (CDCl_3_, 75 MHz): δ 34.0, 45.9, 53.5, 54.9, 62.8, 81.5, 110.5, 125.0, 127.9, 128.4, 128.5, 158.2. LRMS (EI) *m/z* (rel %): 266 (8) [M^+^], 113 (97), 70 (100). HRMS (ESI+) for C_14_H_19_ClN_2_O [M+H]^+^: calcd 267.1259; found 267.1231.

1-methyl-4-[(5-methyl-2,3-dihydro-1-benzofuran-2-yl) methyl]piperazine (**1f**). Yellow oil. ^1^H NMR (CDCl_3_, 300 MHz): δ 2.26 (s, 3H), 2.29 (s, 3H), 2.49 (br.s, 4H), 2.55 (dd, 1H, *J* = 13.4, 4.2 Hz), 2.62 (br.s, 4H), 2.78 (dd, 1H, *J* = 13.4, 7.7 Hz), 2.90 (dd, 1H, *J* = 15.5, 8.0 Hz), 3.22 (dd, 1H, *J* = 15.5, 9.0 Hz), 4.86–4.99 (m, 1H), 6.67 (d, 1H, *J* = 8.1 Hz), 6.89 (d, 1H, *J* = 7.9 Hz), 6.96 (s, 1H). ^13^C NMR (CDCl_3_, 75 MHz): δ 20.8, 34.3, 46.1, 53.8, 55.0, 63.2, 80.8, 109.1, 125.5, 126.5, 128.3, 129.6, 157.4. LRMS (EI) *m/z* (rel %): 245 (25) [M^+^], 113 (100), 70 (85). HRMS (ESI+) for C_15_H_22_N_2_O [M+H]^+^: calcd 247.1806; found 247.1817.

1-methyl-4-[(5-methoxy-2,3-dihydro-1-benzofuran-2-yl) methyl]piperazine (**1g**). Yellow oil. ^1^H NMR (CDCl_3_, 300 MHz): δ 2.30 (s, 3H), 2.30–2.90 (br.s, 8H), 2.57 (dd, 1H, *J* = 13.2, 3.8 Hz), 2.78 (dd, 1H, *J* = 13.2, 8.1 Hz), 2.94 (dd, 1H, *J* = 15.7, 8.1 Hz), 3.24 (dd, 1H, *J* = 15.7, 9.0 Hz), 3.75 (s, 3H), 4.95 (dq, 1H, *J* = 8.4, 3.8 Hz), 6.64 (dd, 1H, *J* = 8.6, 2.3 Hz), 6.71 (d, 1H, *J* = 8.6 Hz), 6.75 (d, 1H, *J* = 2.3 Hz). ^13^C NMR (CDCl_3_, 75 MHz): δ 34.7, 46.1, 53.8, 54.9, 56.0, 63.3, 80.8, 109.5, 111.2, 112.6, 127.5, 153.6, 153.9. LRMS (EI) *m/z* (rel %): 294 (25) [M^+^], 175 (100), 70 (69). LRMS (EI) *m/z* (rel %): 262 (28) [M^+^], 113 (100), 70 (85). HRMS (ESI+) for C_15_H_22_N_2_O_2_ [M+H]^+^: calcd 263.1754; found 263.1755.

1-methyl-4-[(5-tert-butyl-2,3-dihydro-1-benzofuran-2-yl) methyl]piperazine (**1h**). Yellowish solid. mp 90–93°C. ^1^H NMR (CDCl_3_, 300 MHz): δ 1.29 (s, 9H), 2.30 (s, 3H), 2.50 (br.s, 4H), 2.58 (dd, 1H, *J* = 13.4, 4.2 Hz), 2.64 (br.s, 4H), 2.81 (dd, 1H, *J* = 13.4, 7.8 Hz), 2.95 (dd, 1H, *J* = 15.5, 8.1 Hz), 3.27 (dd, 1H, *J* = 15.5, 9.1 Hz), 4.96 (dq, 1H, *J* = 8.3, 4.1 Hz), 6.72 (d, 1H, *J* = 8.1 Hz), 7.14 (d, 1H, *J* = 8.5 Hz), 7.19 (s, 1H). ^13^C NMR (CDCl_3_, 75 MHz): δ 31.8, 34.3, 34.5, 46.0, 53.8, 55.0, 63.4, 80.9, 108.8, 121.9, 124.7, 126.0, 143.4, 157.3. LRMS (EI) *m/z* (rel %): 288 (14) [M^+^], 113 (100), 70 (65). HRMS (ESI+) for C_18_H_28_N_2_O [M+H]^+^: calcd 289.2274; found 289.2263.

1-[(5-chloro-2,3-dihydro-1-benzofuran-2-yl)methyl]-4-phenylpiperazine (**1i**). Yellow oil. ^1^H NMR (CDCl_3_, 300 MHz): δ 2.63 (dd, 1H, *J* = 13.4, 4.2 Hz), 2.75 (q, 4H, *J* = 4.4 Hz), 2.83 (dd, 1H, *J* = 13.4, 7.8 Hz), 2.98 (dd, 1H, *J* = 15.7, 7.8 Hz), 3.20–3.32 (m, 5H), 5.02 (m, 1H), 6.70 (d, 1H, *J* = 8.4 Hz), 6.71 (d, 1H, *J* = 8.4 Hz), 6.86 (dt, 1H, *J* = 7.1, 0.9 Hz), 6.91–6.95 (m, 2H), 7.03–7.08 (m, 1H), 7.10–7.14 (m, 1H), 7.23–7.30 (m, 2H). ^13^C NMR (CDCl_3_, 75 MHz): δ 34.3, 48.0, 53.3, 61.9, 79.7, 110.6, 116.7, 120.9, 125.1, 125.8, 127.5, 128.2, 129.3, 150.3, 157.6. LRMS (EI) *m/z* (rel %): 328 (8) [M^+^], 175 (100), 70 (72). HRMS (ESI+) for C_19_H_21_ClN_2_O [M+H]^+^: calcd 329.1415; found 329.1413.

### Cell Culture and Transfection

HEK293T cells were cultured in DMEM supplemented with 10% fetal bovine serum, 100 U/ml penicillin/streptomycin, at 37°C in 5% CO_2_; 48 h before the binding assays, cells seeded in 10-cm dishes were transiently transfected with H_3_R or H_4_R using polyethylenimine (PEI; 25 kDa linear; Polysciences, Warrington, PA, United States) at a ratio of 3:1 PEI/DNA. For BRET assays, cells were transfected in suspension, using the same PEI/DNA ratio as for attached cells, and directly seeded in 96-well white plates (OptiPlate; PerkinElmer) at a density of 4 × 10^4^ cells/well and grown for 48 h at 37°C in 5% CO_2_. When needed, total DNA amount was adjusted with salmon sperm DNA (Invitrogen, Carlsbad, CA, United States).

### Binding Assays

Competition binding assays were performed in HEK293T cells transiently expressing the H_3_R or H_4_R (Santos et al., 2015) in order to assess the affinities of synthesized compounds for each receptor. HEK293T cells transiently expressing H_3_R or H_4_R were transferred to 24-well culture plates 24 h after the transfection. One day after plating, the cells were washed once with cold wash buffer (Tris–HCl buffer 25 mM, pH 7.4 containing NaCl 140 mM, MgCl_2_ 5 mM, and 0.1% bovine serum albumin). Cells were incubated with 1.15 nM [^3^H]-histamine and increasing concentrations of non-radioactive histamine and compounds as a competitor in cold binding buffer [Tris–HCl 25 mM, pH 7.4, including MgCl_2_ 5 mM, 0.1% bovine serum albumin, and 100 μg/mL bacitracin (Sigma–Aldrich, St. Louis, MO, United States)]. Cells were maintained at 4°C for at least 16 h, washed twice and then lysed with lysis buffer (48% urea, 2% NONIDET P-40, acetic acid 3M). Cell lysates were transferred to scintillation vials and 3 mL of scintillation liquid (ScintiSafe^TM^ Econo 1, Fisher Scientific, Santa Clara, CA, United States) were added. Bound radioactivity was quantified on a Tri-Carb 20100TR liquid scintillation counter (PerkinElmer, Waltham, MA, United States). The median inhibitory concentration (IC_50_) of the tested compounds for displacement [^3^H]-histamine was obtained from the concentration–response curves, and then using the Cheng–Prusoff equation, the apparent affinities [*K*_i_ = IC_50_/(1 + [ligand]/*K*_d_)] were calculated (Cheng and Prusoff, 1973; Reis et al., 2007). Commercially available compounds JNJ-7777120 and clobenpropit were used as reference ligands of H_4_R and H_3_R, respectively.

### Analysis of G Protein Activation by BRET Assays

G protein activation was evaluated in HEK293T cells transiently expressing H_3_R or H_4_R and a BRET-based biosensor composed of Gα_i_ fused to RLucII (in this case, G_i1_-RLucII) and GFP10-Gγ2, in the presence of the untagged Gβ_1_ subunit (Galés et al., 2005). Cells were transfected in suspension, seeded in 96-well white plates (OptiPlate; PerkinElmer) and grown for 48 h. BRET values were monitored using Victor^TM^ X Light Luminescence microplate reader (PerkinElmer) equipped with BRET400-GFP2/10 filter set (acceptor, 515 ± 20 nm; and donor, 400 ± 70 nm filters), 5 min after the addition of 2.5 μM of coelenterazine 400-a (Biotium, Hayward, CA, United States) and the analyzed ligands. In the antagonism assays, the different ligands were incubated 30 min prior to stimulation with histamine and addition of coelenterazine, according to the procedure described earlier.

### Calculated Physicochemical Properties

The molecular properties clogP (calculated lipophilicity), logS (water solubility), molecular weight (MW), topological polar surface area (TPSA) as well as the hydrogen-bond donor (HBD) and acceptor (HBA), and the rotatable bonds (RotB) counts were calculated using the MolSoft online software for drug-likeness and molecular property prediction (La Jolla, CA, United States). The drug-likeness score was also calculated by the software as a prediction of the overall drug-likeness of the molecule based on a MolSofts’s chemical fingerprint. Positive values mean similarity to market drugs.

### Ligand Metric Analyses

The metric analysis of a test molecule in a given target can be done using values of ligand efficiency (LE), lipophilic ligand efficiency (LLE), ligand efficiency dependent lipophilicity (LELP), and group efficiency (GE) (Verdonk and Rees, 2008; Hopkins et al., 2014). These metric values are widely employed in drug discovery to indicate whether a potency value derives from a specific target interaction or simply due to many unspecific contacts. These values were calculated using the following equations (1)–(4).

(1) $$\text{LE}=1.37.\text{p}K_{\text{i}}/\text{HA}$$

(2) $$\text{LLE}=pK_{i}-Clog\text{P}$$

(3) LELP=ClogP/LE

(4) $$\text{GE}=(1.37.\Delta \text{p}K_{i})/\Delta \text{HA}$$

Where *HA* is the number of heavy (non-hydrogen) atoms in the molecule.

### Animals

Male C57Bl/6 mice weighing 20–25 g, 6–8 weeks old, from our own animal facilities were housed in a room with a 12 h light–dark cycle with water and food *ad libitum*. Animal care and research protocols were in accordance with the principles and guidelines adopted by the Brazilian College of Animal Experimentation (COBEA) and this project was approved by the Ethical Committee for Animal Research of the Federal University of São Paulo (CEUA 1666/99).

### Induction of Allergic Asthma and Treatments

Mice (*n* = 5 per group) were sensitized on days 0 and 7 by an intraperitoneal injection of a mixture containing 50 mg of ovalbumin (OVA; Grade V, Sigma–Aldrich, United States) and 1 mg of Al(OH)_3_ in PBS (a total volume of 0.2 ml). Mice were challenged by exposure to an aerosol of OVA generated by an ultrasonic nebulizer (ICEL US-800, SP, Brazil) delivering particles of 0.5–10 mm diameter at approximately 0.75 cc/min for 20 min at days 14 and 21. The concentration of OVA in the nebulizer was 2.5% w/v in PBS. The control group consisted of animals immunized as previously described and challenged two times with PBS solution. Compounds **1d** and **1e** were administered intraperitoneally in two different dosages (5 and 3 mg/kg) to the test groups 30 min before the antigen challenge. Sensitized and control groups were used to assess the anti-inflammatory activity (Corrêa et al., 2017).

### Bronchoalveolar Lavage

Mice were euthanized with intraperitoneal ketamine and xylazine (100 and 10 mg/kg, respectively; Agibrands do Brazil, São Paulo, São Paulo, Brazil), 24 h after exposure to the last aerosol challenge. A tracheal cannula was inserted via a mid cervical incision, and the airways were washed twice with 1 ml of phosphate-buffered saline (PBS, pH 7.4 at 4°C).

### Total and Differential Cell Counts in the Bronchoalveolar Lavage Fluid

The bronchoalveolar lavage fluid was centrifuged at 170 × *g* for 10 min at 4°C, the supernatant was removed, and the cell pellet was re-suspended in 1 ml of PBS. One volume of a solution containing 0.5% crystal violet dissolved in 30% acetic acid was added to nine volumes of the cell suspension. The total number of cells was determined by counting in a hematocytometer. Following cytocentrifugation of the bronchoalveolar lavage fluid, cells were stained with hematoxylin-eosin (Hema 3) for determination of the differential cell numbers (Corrêa et al., 2017).

### Statistical Analyses

The results from *in vivo* evaluations are described as the means ± SEM. Statistical evaluation of the data was carried out using the one-way analysis of variance (ANOVA) followed by Tukey’s post-test. A *p*-value that was lower than 0.05 was considered to be significant. All statistical analyses were performed with the aid of GraphPad software (San Diego, CA, United States).

## Results and Discussions

The design of LINS01 molecules was done considering the presence of an aromatic nucleus (aryl or heteroaryl group) linked to a polar moiety (imidazole, pyrrolidine, or piperazine) observed in H_3_R and H_4_R ligands. The presence of an extra lipophilic group attached to the polar moiety was also evaluated. In a previous paper from Fernandes et al. (2011), a QSAR study defined the importance of the R2 group for the affinity for H_4_R in indolecarboxamides (e.g., chlorine in JNJ-7777120). The role of the R2 group is also important for ligand affinity to H_3_R, which are represented by the cyclopropyl ketone and 4-cyanophenyl groups in H_3_R ligands such as ciproxifan and ABT-239, respectively (Cowart et al., 2004). Considering this, the present set of molecules includes the substitutions not only in piperazine nitrogen (R1), but also in the dihydrobenzofuran moiety (R2) to determine preliminary SAR data for these compounds. The results show that the R2 substituents can play an important role in the binding affinities, being possibly more important than the substituent R1 to drive the receptor selectivity.

The 2-allylphenol was used as a starting material (**Figure 2**) for compounds **1a**–**d**, which was converted into the (2-iodomethyl)-2,3-dihydrobenzofuran, as previously described by our group, giving excellent yield (**Table 1** and Supplementary Information) (Corrêa et al., 2016, 2017). The iodinated dihydrobenzofuran **4a** was then reacted with the corresponding piperazines in considerable excess, giving moderate to good yields. The yields of this step are presented in **Table 1**. A similar approach was applied to prepare the commercially unavailable 1-allylpiperazine **5** (Supplementary Figure S1) with 40% yield. The 1-methyl and 1-phenyl piperazines are commercially available.

The compounds **1e**–**i** were prepared starting from the corresponding 4-substituted phenol, which was allylated using allyl bromide, and then thermally isomerized through Claisen rearrangement to give the corresponding 2-allylphenols, as reported previously by Corrêa et al. (2017). Both steps resulted in good to excellent yields (**Table 1** and Supplementary Information). With the 4-substituted 2-allylphenols, the final compounds were prepared in the same fashion to compounds **1a**–**d** (Corrêa et al., 2016).

**FIGURE 3:**
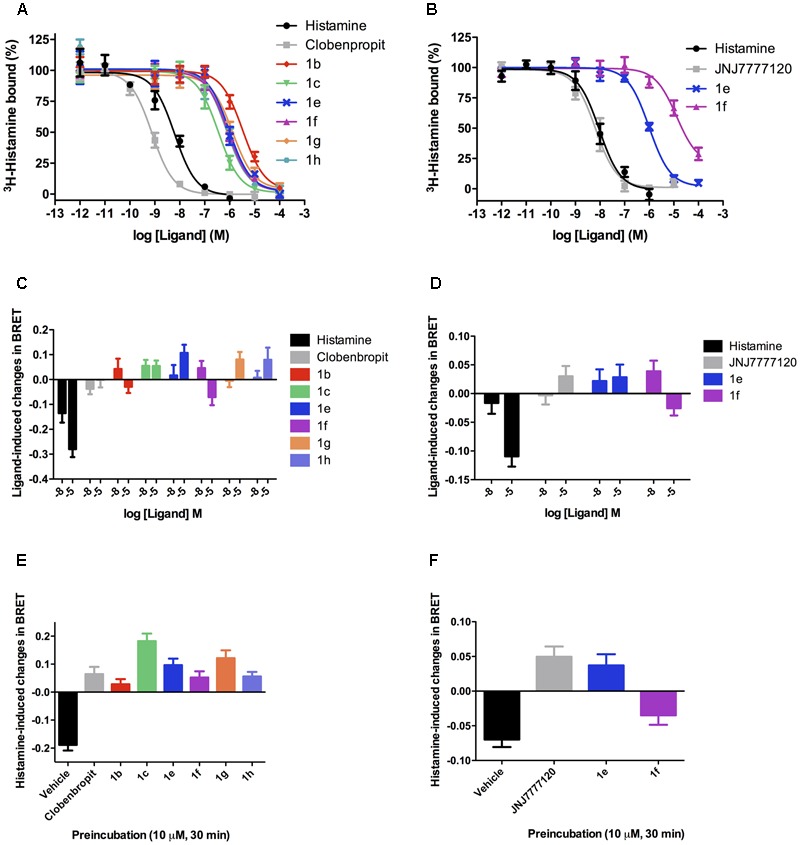
Binding and functional characterization of the compounds in the H_3_R and H_4_R. Competition dose–response binding curves for selected compounds show that they presented lower affinity for either H_3_R **(A)** or H_4_R **(B)**, as compared with histamine and selective ligands, with *K*_i_ values varying from high nanomolar to low micromolar. Functional assays in the H_3_R **(C)** reveal that all compounds do not trigger G_i_ activation, suggesting that they may behave as antagonists in this receptor. In the H_4_R **(D)** a similar profile was observed. Antagonistic assays show that all tested compounds when preincubated at 10,000 nM were able to fully block histamine activity in the H_3_R **(E)** and H_4_R **(F)**, except for compound **1f** that only partially blocked histamine activity on H_4_R, probably due to a weak agonistic effect in high concentrations. Data were obtained from at least three independent experiments performed in duplicate. It is also interesting to note that either on H_3_R or on H_4_R some compounds displayed a profile that suggests a mild inverse agonist activity, as can be observed by an inverse value of BRET as compared to histamine.

The binding analyses were initially performed in the format of a screening, to allow a quick assessment of the compounds presenting relevant affinity for H_3_R and/or H_4_R (Supplementary Figure S2). The results show that most of the compounds presented higher affinity for H_3_R, and that only compounds **1e** and **1f** displayed considerable binding to H_4_R. Accordingly, competition binding assays with full concentration–response curves were performed with the selected compounds in the H_3_R and H_4_R (**Figure 3**). The obtained *K*_i_ values are in the range of 0.4–10 μM (**Table 2**).

**Table 2:** p*K*_i_ values of tested compounds for H_3_R and H_4_R.

Compounds	H_3_R p*K*_i_^a^ ± SEM	H_4_R p*K*_i_^a^ ± SEM	SI^b^ H_4_R/H_3_R (*K*_i_)
Histamine	8.53 ± 0.31	8.21 ± 0.29	
Clobenpropit	9.25 ± 0.15	n.d.	
JNJ-7777120	n.d.	8.25 ± 0.31	
**1a**	<5.0	<5.0	n.d.
**1b**	5.57 ± 0.17	<5.0	>3.7
**1c**	6.40 ± 0.21	<5.0	>25
**1d**	<5.0	<5.0	n.d.
**1e**	6.07 ± 0.07	6.06 ± 0.06	1.1
**1f**	6.15 ± 0.08	5.00 ± 0.32	14
**1g**	6.06 ± 0.04	<5.0	>11
**1h**	6.07 ± 0.10	<5.0	> 11
**1i**	<5.0	<5.0	n.d.

*^a^p*K*i values are presented as mean ± SEM of at least three independent experiments. ^b^Selectivity index. n.d.: *K*i not determined.*

Drug-likeness is a qualitative evaluation of a certain compound for how drug-like it is with respect to some factors such as bioavailability and potency based on the characteristics of market drugs. The drug-likeness of the compounds was assessed through the Lipinski’s rule-of-five (Lipinski et al., 2001) and Veber’s rules (Veber et al., 2002) parameters as well as water solubility. Lipinski’s rule-of-five proposes that a molecule should have MW < 500, clogP < 5, HBD < 5 and HBA < 10 to have good oral bioavailability. Veber’s rules include in these parameters the TPSA < 140 and RotB < 10. The LINS01 compounds fulfill all these parameters (**Table 3**), suggesting they should have good bioavailability and possibly adequate pharmacokinetic profile. The water solubility also determines the bioavailability and other ADME processes. LINS01 molecules show adequate water solubility, as indicated by the logS values (acceptable values for > -4). With this regard, it is noteworthy the contribution of methoxy group (**1g**) to enhanced water solubility. The balanced hydrophilic–lipophilic character of LINS01 molecules (as indicated by logP < 5 and logS > -4 values) suggests they may have good pharmacokinetic behavior *in vivo*. Drug-likeness scores corroborate to this estimation, since only positive values were obtained (**Table 3**). Considering that market drugs and non-drugs usually present, respectively, positive and negative score values, LINS01 molecules can be considered promising drug-like compounds to further optimization according to this criteria.

**Table 3:** Drug-likeness calculations for the compounds 1a–1i.

Compounds	cLogP	LogS	TPSA	HBA	HBD	RotB	Drug-likeness score^a^
**1a**	1.30	-1.23	24.50	3	1	2	0.86
**1b**	1.90	-1.20	15.71	3	0	2	1.21
**1c**	2.54	-2.26	15.71	3	0	4	0.90
**1d**	3.59	-2.63	15.71	3	0	3	1.19
**1e**	2.73	-2.05	15.71	3	0	2	1.49
**1f**	2.32	-1.48	15.71	3	0	2	1.10
**1g**	1.93	-1.31	24.94	4	0	3	1.33
**1h**	3.58	-2.94	15.71	3	0	3	1.09
**1i**	4.25	-3.48	15.71	3	0	3	1.35

*^a^The score was calculated using the MolSoft software algorithm.*

In order to evaluate the potency of these compounds, metric LE, LLE, LELP, and GE values were calculated (**Table 4**). During drug discovery and development process, the MW and lipophilicity usually increase in consonance with the potency. This can be explained by the contribution of the inserted non-hydrogen atoms to binding energy. LE is considered a weighted value for non-hydrogen atoms contributions to binding energy, which allows to compare affinities of molecules with different sizes (Hopkins et al., 2014). Ligands with LE > 3 are considered promising drug-like compounds. For comparison, the mean value for oral drugs is around 4.5. Considering that lipophilicity also influences on binding affinity, the LLE and LELP values are lipophilicity-weighted LE values. The ideal LLE value for an optimized drug candidate is ∼5–7, while LELP optimal range values are between -10 and 10. GE is a measurement of the binding efficiency for an added functional group in the parent molecule (Verdonk and Rees, 2008). Accordingly, it represents how much the added group contributed to overall affinity of the molecule, considering the number of heavy atoms. For example, a GE = 0.31 should represent at least a gain of 1.7-fold in the potency of a small molecule (MW < 500). The higher the GE value, the higher the contribution of the group to the potency.

**Table 4:** Ligand metric analysis of the compounds tested for H_3_R and H_4_R.

Compounds	H_3_R				H_4_R			
	LE	LELP	LLE	GE	LE	LELP	LLE	GE
**1b**	0.45	4.23	3.67	> 0.78^a^				
**1c**	0.46	5.50	3.86	>0.63^a^				
**1e**	0.46	5.90	3.34	0.69^b^	0.46	5.92	3.33	>1.45^b^
**1f**	0.47	4.96	3.83	0.80^b^	0.38	6.08	2.69	-
**1g**	0.44	4.42	4.13	0.34^b^				
**1h**	0.40	9.04	2.49	0.17^b^				

*^a^GE of the group inserted on R1 of **1a**. ^b^GE of the group inserted on R2 of **1b**.*

Regarding the H_3_R, the *N*-allyl derivative **1c** showed the highest affinity in the series, showing a p*K*_i_ value of 6.40, followed by the chloro, methoxy, and *t-*butyl derivatives **1e**, **1g**, and **1h**, with p*K*_i_ values around 6.07, and the methyl derivative **1f** with p*K*_i_ 6.15. The non-alkylated piperazine compound **1a**, as well as the *N*-phenyl derivatives **1d** and **1i** did not show appreciable affinity for H_3_R, as mentioned before. The binding efficiencies of the compounds **1b**, **1c**, and **1e–h** are considered adequate, since they present LE > 3 and with exception of compounds **1g** and **1h**, ≥4.5, indicating that although their low MW, their efficiencies are comparable to a real drug. LELP and LLE values suggest that lipophilicity is relatively high in these molecules, thus suggesting that polar groups (which can improve binding affinity) can be inserted for improvement in a future set of compounds. We believe that H_3_R may have a considerable different binding pocket than H_4_R in the R1 and R2 regions, which allow the interaction of bulky hydrophobic groups (such as allyl in R1 and *t-*butyl in R2). However, some steric hindrance effects might also play a role (especially in the R1 region), limiting the interaction of bulkier groups such as the phenyl group of **1d** or **1i**, but allows the interaction of the allyl group present in **1c**. The results also suggest that the presence of an alkyl group linked to the polar moiety of the molecule increases the affinity for H_3_R, as can be seen by the high GE values for methyl (**1b**) and allyl (**1c**) groups (**Table 4**).

Several 2-aminoethylbenzofurans have already been evaluated as H_3_R ligands, showing good affinity (Cowart et al., 2004). These compounds can be viewed as rigid analogs of ciproxifan, such as ABT-239. Hence, the compounds here presented can also be considered rigid derivatives of ciproxifan, but lacking aromaticity in the furan ring. As observed in ABT-239 analogs, small alkyl substituents in the polar moiety increase H_3_R affinity. This could be the explanation for the poor affinity of **1a** and moderate affinities for **1c** and the *N*-methyl derivatives **1e–h** (**Figure 3** and **Table 2**).

Since ABT-239 and its analogs possess a 5-aryl substituted benzofuran and better affinity, substituents at the 5-position were explored to evaluate the role of the R2 group in the dihydrobenzofuran derivatives. These data (**Table 2**) define the importance of the R2 substituent in the binding affinity to H_3_R of these compounds. Although there are no significant differences between the affinity of compounds **1e–h** to H_3_R, the affinity gain caused by the R2 substitution can be noted by the p*K*_i_ of the non-substituted molecule **1b** (5.57). Although compounds **1e–h** present quite similar p*K*_i_, the GE is very different among the substituents (**Table 4**). Methyl group (**1f**) has shown the higher contribution to binding efficacy in H_3_R, while *t*-butyl group (**1h**) gave lower GE value. The presence of a polar group in R2 (such as methoxy in **1g**) also suggests that hydrophobic groups are preferred in this moiety, as indicated by GE = 0.34.

**FIGURE 4:**
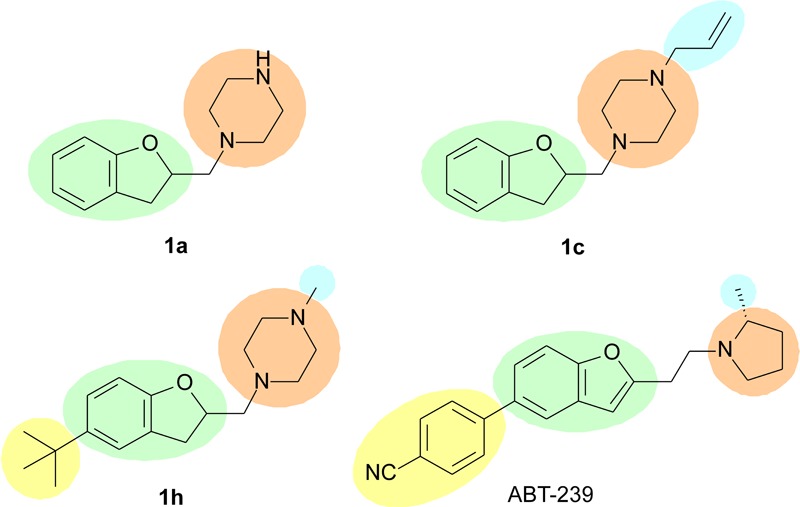
Possible characteristics involved in H_3_R binding; green – aryl or heteroaryl; orange – polar group; blue – lipophilic group; yellow – bulky group. The yellow group may define the selectivity for the H_3_R.

In summary, substitutions in R2 seem to play an important role in H_3_R binding, since substituting the hydrogen in this position led to improved affinity for the receptor. A rationale SAR for the substitution pattern cannot be defined yet, and further substituents must be explored. However, it is possible to verify that possibly larger volume is tolerated in this region of the molecule, such as the *t*-butyl group in **1h**, which is not tolerated when interacting to the H_4_R. Possibly, this group presents the same role than the cyanophenyl group in ABT-239 (**Figure 4**), and maybe it could generate selectivity toward H_3_R. Previous report from Dastmalchi et al. (2008) suggests that bulkier molecules may display increased affinities for H_3_R, possibly because there is a hydrophobic pocket nearby the R2 region which can be accessed by these groups, driving the selectivity toward H_3_R over H_4_R. With exception of compound **1e**, the molecules with substituents in R2 presented over 10-fold selectivity for H_3_R as compared to H_4_R (**Table 2**). Other molecules presenting other bulky groups in R2 are in progress by our group to verify this possibility.

Regarding the affinity for H_4_R, compound **1e** was the only one to present affinity in the micromolar range (p*K*_i_ 6.06). Compound **1f** has a *K*_i_ in the range of ∼10,000 nM. The remaining molecules presented negligible binding affinities, as found in our initial screening approach (see Supplementary Information) and aforementioned. Considering the similarity of the presented compounds with the high affinity H_4_R antagonist JNJ-7777120, it seems that the absence of the carbonyl group in addition to the change of the indole ring to dihydrobenzofuran is detrimental to the affinity of the ligands for H_4_R. In fact, the changes in affinity caused by such changes corroborate previous findings in benzofuran compounds (Engelhardt et al., 2012).

Previous reports (Venable et al., 2005; Engelhardt et al., 2012) suggested that a methyl group is the best substituent in the piperazine nitrogen of indolecarboxamide and benzimidazol carboxamide series. Higher homologs or bulkier groups lead to molecules with lower affinity for H_4_R. In the present series, this pattern was also detected, since compounds **1e** and **1f** are *N*-methyl derivatives. The LE values suggest these molecules present good efficacy in the H_4_R binding affinity. Moreover, LELP and LLE values also suggest that **1e** and **1f** represent drug-like molecules that can still be optimized regarding the hydrophilic–lipophilic balance. The present results suggest the R2 substituent is more important to the binding affinity than the R1 group, and bulkier groups (such as phenyl in **1d** and **1h**) lead to low binding affinities (>10,000 nM) when binding H_4_R. Venable et al. (2005) evaluated several H_4_R ligands with different substituents in R2 position, with chlorine being the group leading to an important increase in the affinity. It is proposed that this atom performs a key interaction with the binding pocket of H_4_R. In the present report, this role was also observed, since compound **1e** presented the highest affinity in the series for this receptor. The insertion of the chlorine atom resulted in more than 10-fold increase in affinity and a very high GE value (**Table 4**). This suggests that chlorine atom yielded in improved efficiency for **1e** as H_4_R ligand. In addition, when methyl is present in R2, the affinity drops significantly, and bulkier groups led to total loss of affinity. Accordingly, the H_4_R seems to present steric hindrance in the R2 region, which is not observed to H_3_R.

The functional activities of the LINS01 compounds were performed to evaluate their possible actions as agonists of H_3_R and/or H_4_R. Both H_3_R and H_4_R are GPCRs known to be G_i_-coupled. To determine the possible activity of the compounds as agonists of this pathway, we used a BRET biosensor to assess and quantify G_i_1 isoform activation.

Regarding H_3_R, **Figure 3C** shows that all analyzed compounds were unable to trigger G_i_ activation even in the highest tested concentration (10,000 nM), likewise clobenpropit, a known H_3_R antagonist. On the other hand, histamine (used as a positive control) yielded a strong activation signal at 10 and 10,000 nM. The results suggest that the compounds could act as antagonists and block the activation triggered by histamine. This hypothesis was indeed confirmed when cells expressing H_3_R were incubated with the LINS01 compounds prior to stimulation with histamine. As can be seen in **Figure 3E**, preincubation with any of the tested compounds at the concentration of 10,000 nM fully blocked histamine agonistic effect. The compounds were equally efficacious in antagonizing histamine effects when compared to clobenpropit, and therefore can be considered H_3_R antagonists. Interestingly, some compounds displayed a profile of reversal of BRET values, as compared to histamine alone, suggesting an inverse agonist activity.

Analyses of functional activity on H_4_R showed that similarly to JNJ-777120, a known H_4_R antagonist, the chlorinated compound **1e** did not trigger G_i_ activation (**Figure 3D**). Possibly the specific interaction of chlorine with H_4_R does not lead to activation of the receptor, but when methyl group is present an agonist mode interaction may occur and therefore a halogen-bonding interaction is suggested rather than a hydrophobic interaction to reach the antagonist binding mode. The role of chlorine in H_4_R ligands was already widely studied, and it has been directly linked to the antagonist activity on G_i_ as well as β-arrestin activation (Nijmeijer et al., 2013). It is important to stress that histamine itself was a poor stimulant of G_i_ in H_4_R (compare BRET values from **Figures 3C,D**). When evaluating the potential to block histamine activity, preincubation with the compound **1e** at the concentration of 10,000 nM fully blocked histamine agonistic effect, similarly to the effect obtained for the H_4_R antagonist JNJ-777120. The compound **1f**, as described above, displays a moderate agonistic effect, and therefore only partially reduced histamine effect.

**FIGURE 5:**
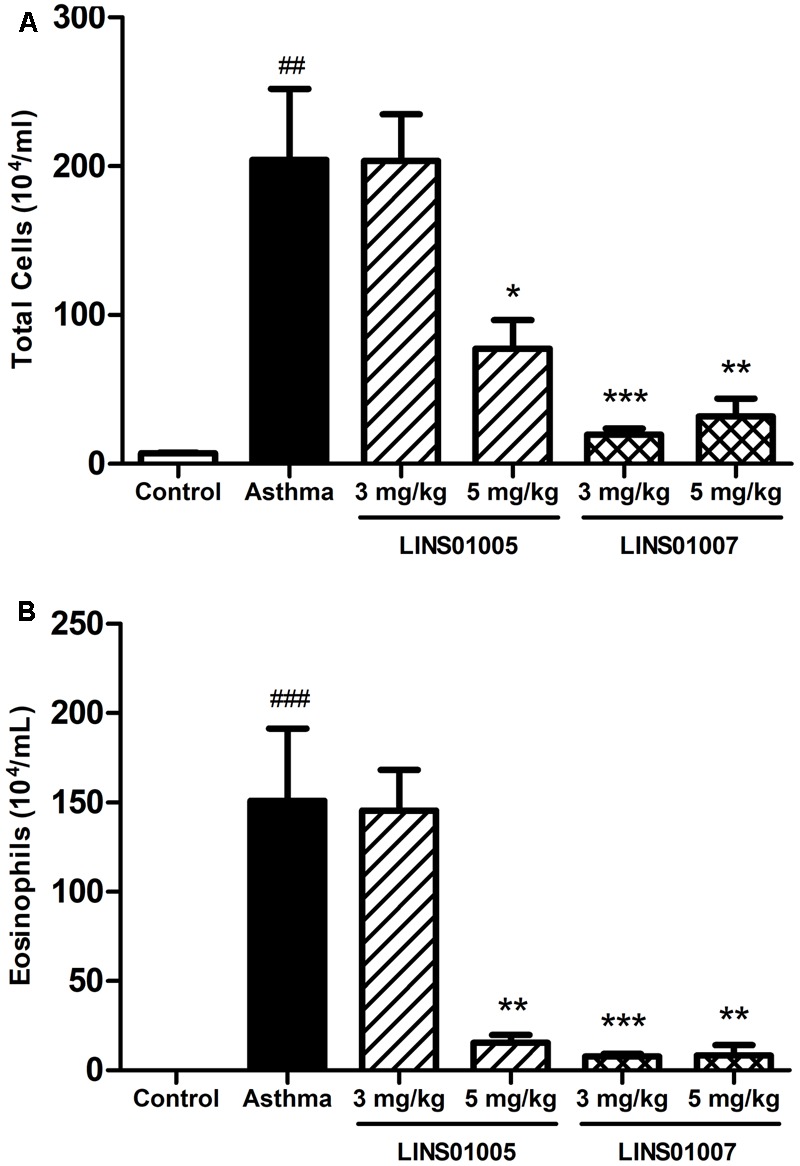
Effects of compounds **1d** (LINS01005) and **1e** (LINS01007) in the **(A)** total inflammatory cell count and **(B)** eosinophil count obtained from bronchoalveolar lavage (3 and 5 mg/kg); ^##^*p* < 0.01 relative to control group; ^∗^*p* < 0.05, ^∗∗^*p* < 0.01, and ^∗∗∗^*p* < 0.001 relative to asthma group.

In order to evaluate the anti-inflammatory potential of the best H_4_R blocker from the LINS01 set, compound **1e** was tested in a mouse asthma model and compared to the previously tested compound **1d** in two different doses (3 and 5 mg/kg, **Figure 5**). Experimental murine asthma was induced by sensitization and provocation with OVA. In a recent report (Corrêa et al., 2017), we verified that **1d** showed anti-inflammatory activity only at 5 mg/kg dose.

In the present work, we evaluated in parallel the compounds **1e** (LINS01007) and **1d** (LINS01005) to rodents. The reduction in cell counts in both groups correlates well to the affinity to H_4_R (see **Figure 3B**), supporting the involvement of this receptor in the mechanism of the observed anti-inflammatory action. The results presented in **Figure 5** also suggest a dose-dependent effect for compound **1d** on both total cell and eosinophil counts in bronchoalveolar lavage fluids, since a sounder reduction in cellularity was observed in the highest concentration of 5 mg/kg. This reduction can be considered more relevant due to the important reduction in eosinophilia. On the other hand, compound **1e** led to low counts in both doses. Possibly even in the lower dose (3 mg/kg) the maximum efficacy was obtained.

In bronchoalveolar lavage fluids with experimental allergic asthma, enhanced cell counts are commonly observed. In particular, there is considerable increase in eosinophils, which are commonly absent or in low density in the non-sensitized animals. The involvement of H_4_R in experimental murine asthma model has been demonstrated in several studies, including results with JNJ-7777120, which was capable of reducing the inflammatory infiltrations (especially eosinophils) and other asthma parameters (Neumann et al., 2013). Considering that the pharmacological profile obtained with LINS01 compounds in the total cell and eosinophil counts is very similar to that obtained with the JNJ compound, we believe the infiltration reductions are correlated to H_4_R. Moreover, the higher activity observed to **1e** also supports this conclusion.

## Conclusion

In this study, we present nine LINS01 molecules with quite simple structure, which have been evaluated concerning binding affinities for the H_3_R and H_4_R, generating corresponding receptor subtype selectivity values. We have also performed functional analyses of G_i_ activation, which revealed that all tested compounds act as antagonists to different extents on both H_3_R and H_4_R. This is the first report regarding functional activity of such compounds. SAR information concerning this chemotype was generated and metric analyses suggest that future derivatives with improved affinity can still be obtained. *In vivo* evaluation in a mouse asthma model was performed and the potential anti-inflammatory activity of such molecules is exemplified by **1e**, which showed to be highly effective at low doses.

## Author Contributions

MC and ÁB performed the synthesis of the compounds, and were supervised by JF at LINS-UNIFESP. LT, DD, SS, and LP performed the binding and functional assays at CC-N’s laboratory, their supervisor. AB and RL did the *in vivo* assays. MB generated the constructions for BRET assays and discussed obtained data. JF and CC-N wrote the manuscript.

## Conflict of Interest Statement

The authors declare that the research was conducted in the absence of any commercial or financial relationships that could be construed as a potential conflict of interest.
