# Supplementary material for: Pharmacological Characterization of 5-Substituted 1-[(2,3-dihydro-1-benzofuran-2-yl)methyl]piperazines: Novel Antagonists for the Histamine H3 and H4 Receptors with Anti-inflammatory Potential
Source: Front Pharmacol. 2017 Nov 14;8:825. doi: 10.3389/fphar.2017.00825 (PMC5694482; doi:10.3389/fphar.2017.00825)
Supplement: Supplementary file 1 [file Data_Sheet_1.PDF]

Supplementary Material

Pharmacological characterization of 5-substituted 1-[(2,3-dihydro-1-benzofuran-2-yl)methyl]piperazines: novel antagonists for the histamine H_3 and H_4 receptors with anti-inflammatory activity

Michelle F. Corrêa, Álefe J. R. Barbosa, Larissa B. Teixeira, Diego A. Duarte, Sarah C. Simões, Lucas T. Parreiras-e-Silva, Aleksandro M. Balbino, Richardt G. Landgraf, Michel Bouvier, Claudio M. Costa-Neto*, João Paulo S. Fernandes*

*** Correspondence:** Corresponding Author: claudio@fmrp.usp.br (CMCN); joao.fernandes@unifesp.br (JPSF).

1 Supplementary Data

1-Allyloxi-4-chlorobenzene (**2e**). Colorless oil. ^{1}H NMR ($CDCl_3$, 300 MHz): δ 4.48 (d, 2H, *J* = 5.3 Hz), 5.28 (d, 1H, *J* = 10.5 Hz), 5.39 (d, 1H, *J* = 17.3 Hz), 5.94-6.10 (m, 1H), 6.78-6.86 (m, 2H), 7.17-7.26 (m, 2H). ^{13}C RMN ($CDCl_3$, 75 MHz): δ 69.1, 116.1, 117.9, 125.7, 129.3, 133.0, 157.2.

1-Allyloxy-4-methylbenzene (**2f**). Colorless oil. ^{1}H NMR ($CDCl_3$, 300 MHz): δ 2.28 (s, 3H), 4.51 (dt, 2H, *J* = 5.3, 1.5 Hz), 5.27 (dq, 1H, *J* = 10.5, 1.4 Hz), 5.40 (dq, 1H, *J* = 17.3, 1.4 Hz), 6.05 (ddt, 1H, *J* = 17.3, 10.5, 1.4 Hz), 6.76-6.86 (m, 2H), 7.03-7.11 (m, 2H). ^{13}C RMN ($CDCl_3$, 75 MHz): δ 20.7, 69.8, 114.6, 118.1, 129.5, 130.7, 133.8, 161.0.

1-Allyloxy-4-methoxybenzene (**2g**). Colorless oil. ^{1}H NMR ($CDCl_3$, 300 MHz): δ 3.75 (s, 3H), 4.47 (dt, 2H, *J* = 5.3, 1.5 Hz), 5.26 (dq, 1H, *J* = 10.5, 1.4 Hz), 5.40 (dq, 1H, *J* = 17.3, 1.4 Hz), 6.04 (ddt, 1H, *J* = 17.3, 10.5, 1.4 Hz), 6.78-6.89 (m, 4H). ^{13}C RMN ($CDCl_3$, 75 MHz): δ 55.7, 69.5, 114.6, 115.7, 117.5, 133.7, 152.8, 153.9.

1-Allyloxy-4-(tert-butyl)benzene (**2h**). Colorless oil. ^{1}H NMR ($CDCl_3$, 300 MHz): δ 1.30 (s, 9H, 4.52 (d, 2H, *J* = 5.3 Hz), 5.27 (dq, 1H, *J* = 10.5, 1.4 Hz), 5.40 (dq, 1H, *J* = 17.2, 1.4 Hz), 5.98-6.14 (m, 1H), 6.82-6.90 (m, 2H), 7.25-7.34 (m, 2H). ^{13}C RMN ($CDCl_3$, 75 MHz): δ 31.6, 34.1, 68.9, 114.2, 117.5, 126.2, 133.6, 143.5, 156.4.

2-Allyl-4-chlorophenol (**3e**). Yellowish liquid. ^{1}H NMR ($CDCl_3$, 300 MHz): δ 3.36 (d, 2H, *J* = 6.4 Hz), 5.03 (br.s, 1H), 5.15 (dq, 1H, *J* = 10.6, 1.5 Hz), 5.17-5.22 (m, 1H), 5.97 (ddt, 1H, *J* = 16.8, 10.6, 1.5 Hz), 6.70-6.76 (m, 1H), 7.05-7.12 (m, 1H), 7.09 (s, 1H). ^{13}C RMN ($CDCl_3$, 75 MHz): δ 34.8, 117.0, 117.2, 125.6, 127.3, 127.6, 130.1, 135.5, 152.6.

2-Allyl-4-methylphenol (**3f**). Yellowish liquid. ^{1}H NMR ($CDCl_3$, 300 MHz): δ 2.25 (s, 3H), 3.33-3.39 (m, 2H), 4.96 (br.s, 1H), 5.11 (t, 1H, *J* = 1.6 Hz), 5.16 (dq, 1H, *J* = 6.9, 1.6 Hz), 5.92-6.05 (m, 1H), 6.66-6.72 (m, 1H), 6.88-6.94 (m, 2H). ^{13}C RMN ($CDCl_3$, 75 MHz): δ 20.5, 35.1, 115.7, 116.3, 125.2, 128.3, 130.2, 131.0, 136.6, 151.8.

2-Allyl-4-methoxyphenol (**3g**). Yellowish liquid. ^{1}H NMR ($CDCl_3$, 300 MHz): δ 3.35-3.40 (m, 2H), 3.75 (s, 3H), 4.82 (s, 1H), 5.12 (sext, 1H, *J* = 1.7 Hz), 5.14-5.19 (m, 1H), 6.00 (ddt, 1H, *J* = 17.6, 9.7,

6.3 Hz), 6.64-6.77 (m, 3H). ^{13}C RMN ($CDCl_3$, 75 MHz): δ 35.2, 55.8, 112.7, 116.0, 116.5, 126.6, 136.2, 148.0, 153.8.

2-Allyl-4-(tert-butyl)phenol (**3h**). Yellowish liquid. ^{1}H NMR ($CDCl_3$, 300 MHz): δ 1.29 (s, 9H), 3.41 (d, 2H, *J* = 6.2 Hz), 4.93 (br.s, 1H), 5.11-5.15 (m, 1H), 5.18 (dq, 1H, *J* = 10.5, 1.6 Hz), 5.96-6.11 (m, 1H), 6.74 (d, 1H, *J* = 8.3 Hz), 7.09-7.18 (m, 2H). ^{13}C RMN ($CDCl_3$, 75 MHz): δ 31.6, 35.6, 114.8, 115.4, 116.4, 124.5, 124.7, 127.4, 136.7, 151.8.

1-Allylpiperazine (**5**). Yellow oil. ^{1}H NMR ($CDCl_3$, 300 MHz): δ 2.01 (s, 1H), 2.42 (br, 4H), 2.86 (s, 1H), 2.90 (t, 2H, J = 4.8 Hz), 2.96-3.04 (m, 2H), 5.11-5.23 (m, 2H), 5.87 (ddt, 1H, 17.1, 10.3, 6.6 Hz). ^{13}C RMN ($CDCl_3$, 75 MHz): δ 46.0, 54.4, 62.4, 118.0, 134.9.

2 Supplementary Figures

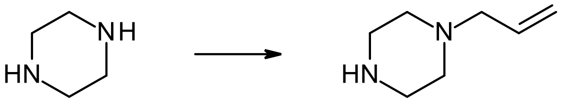

5

Supplementary Figure 1. Reaction scheme for the synthesis of 1-allylpiperazine.

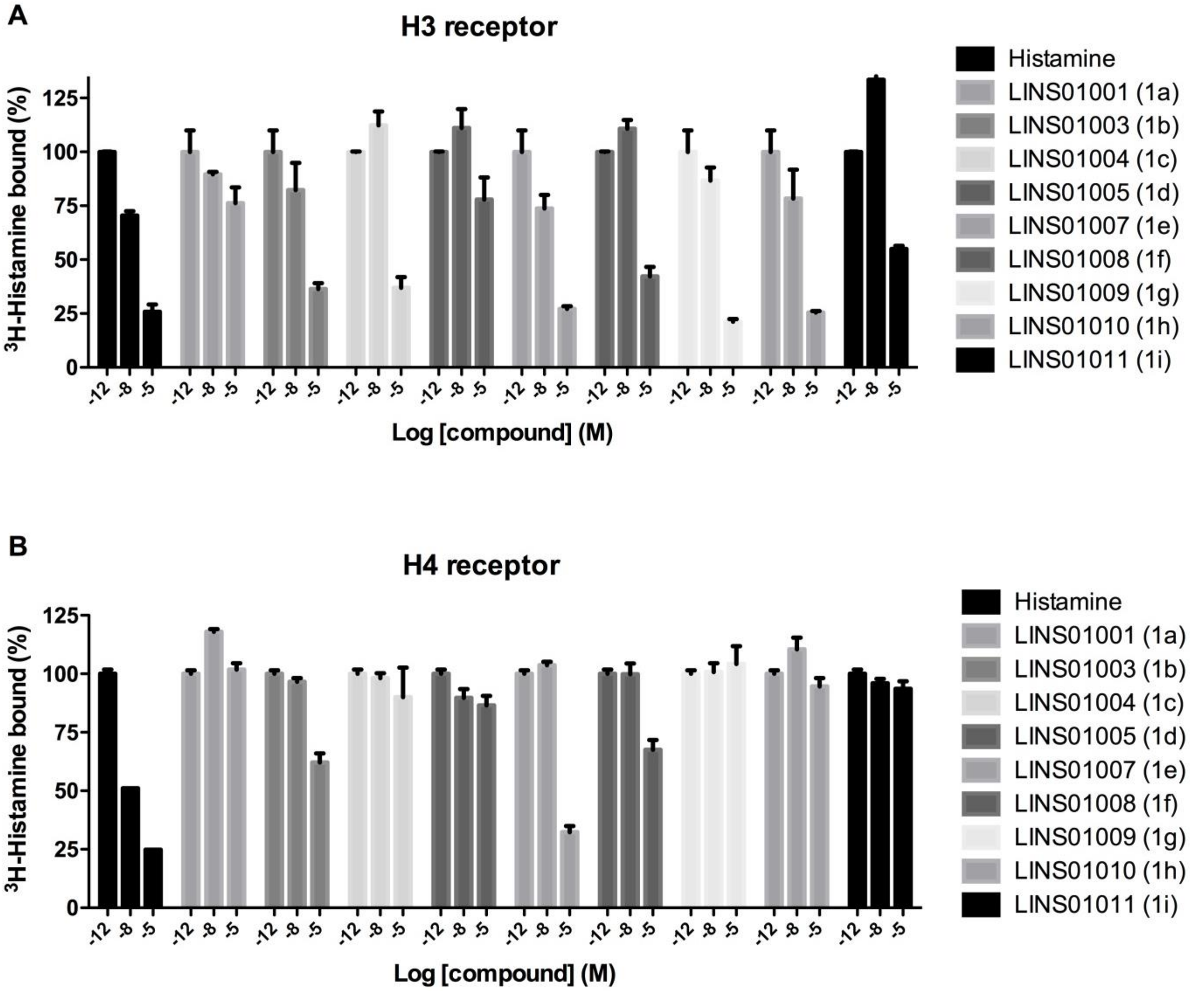

Supplementary Figure 2. Preliminary binding data for the final compounds **1a-h**.

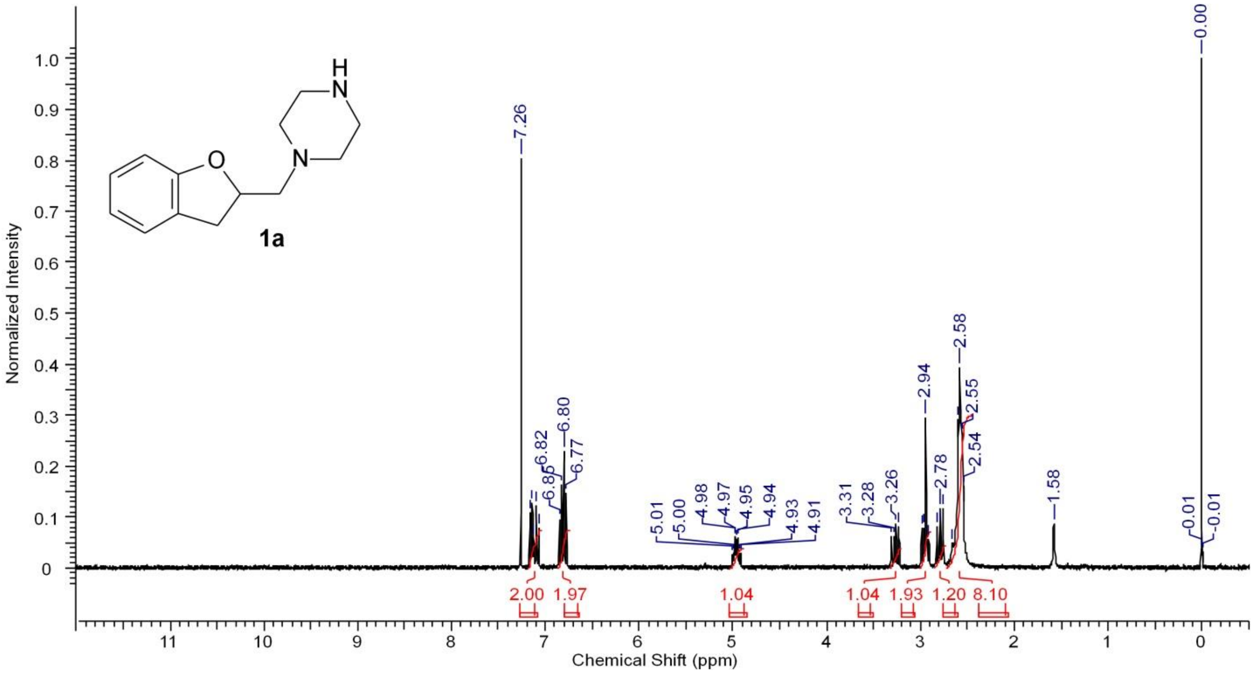

Supplementary Figure 3. Copy of the ^{1}H-NMR spectra of compound **1a**.

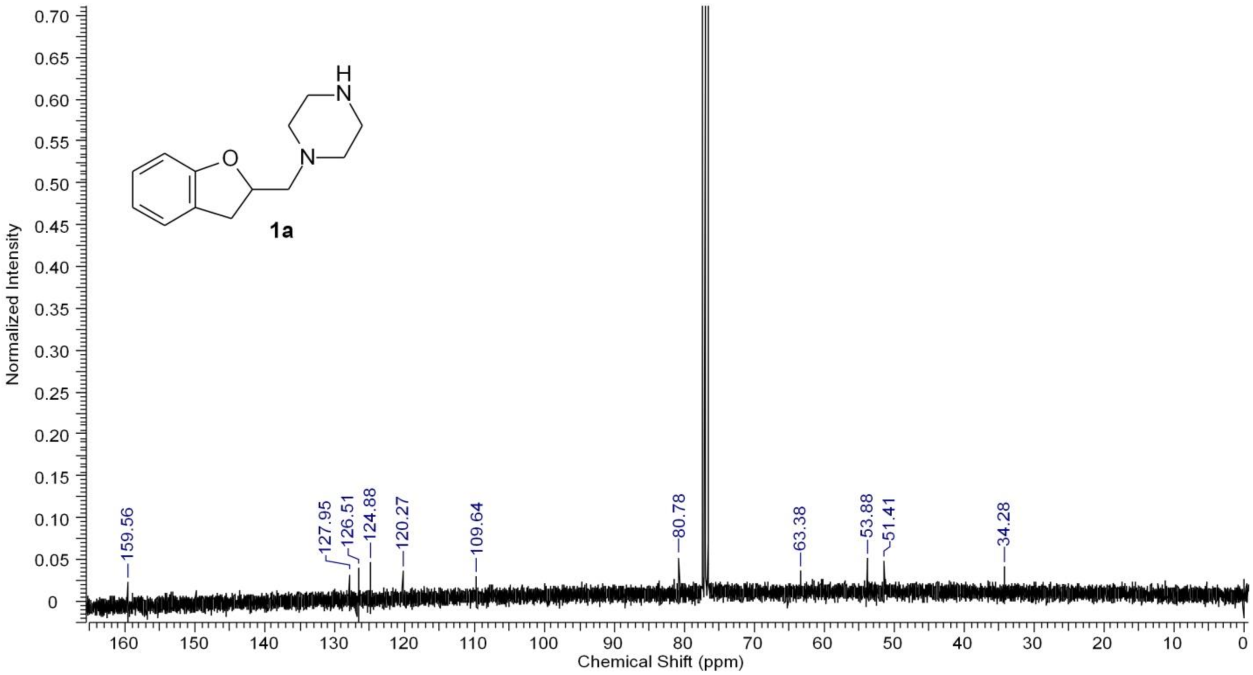

Supplementary Figure 4. Copy of the ^{13}C-NMR spectra of compound **1a**.

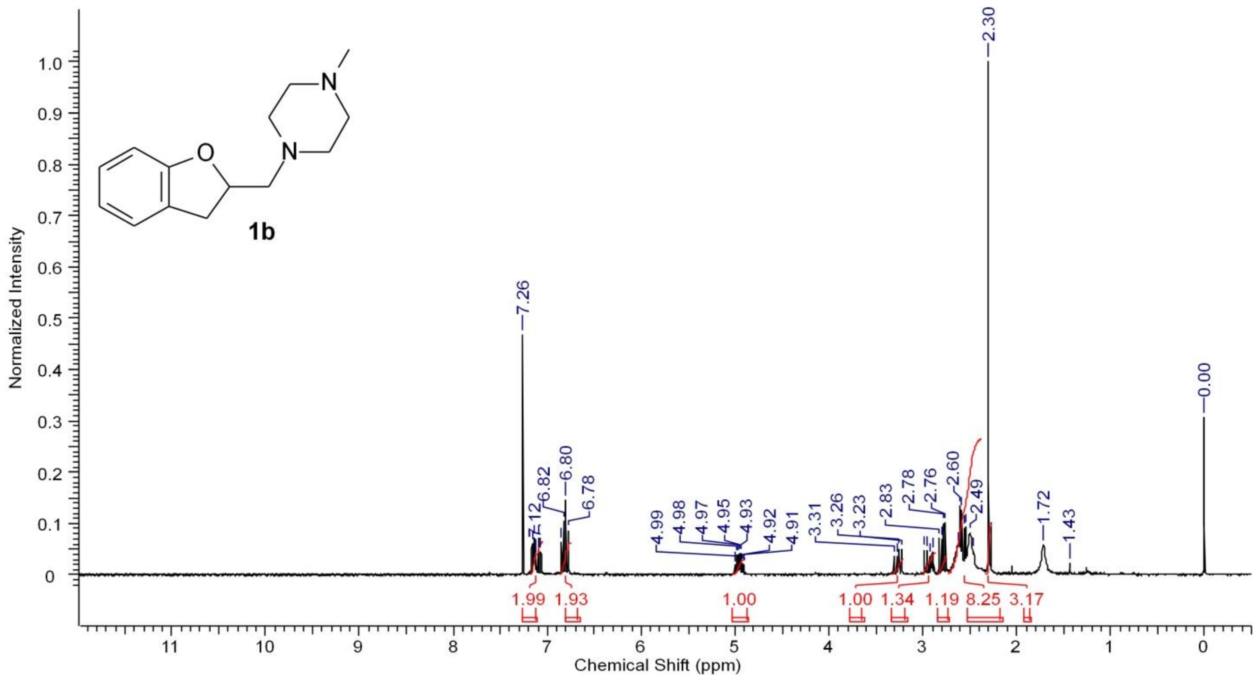

Supplementary Figure 5. Copy of the ^{1}H-NMR spectra of compound **1b**.

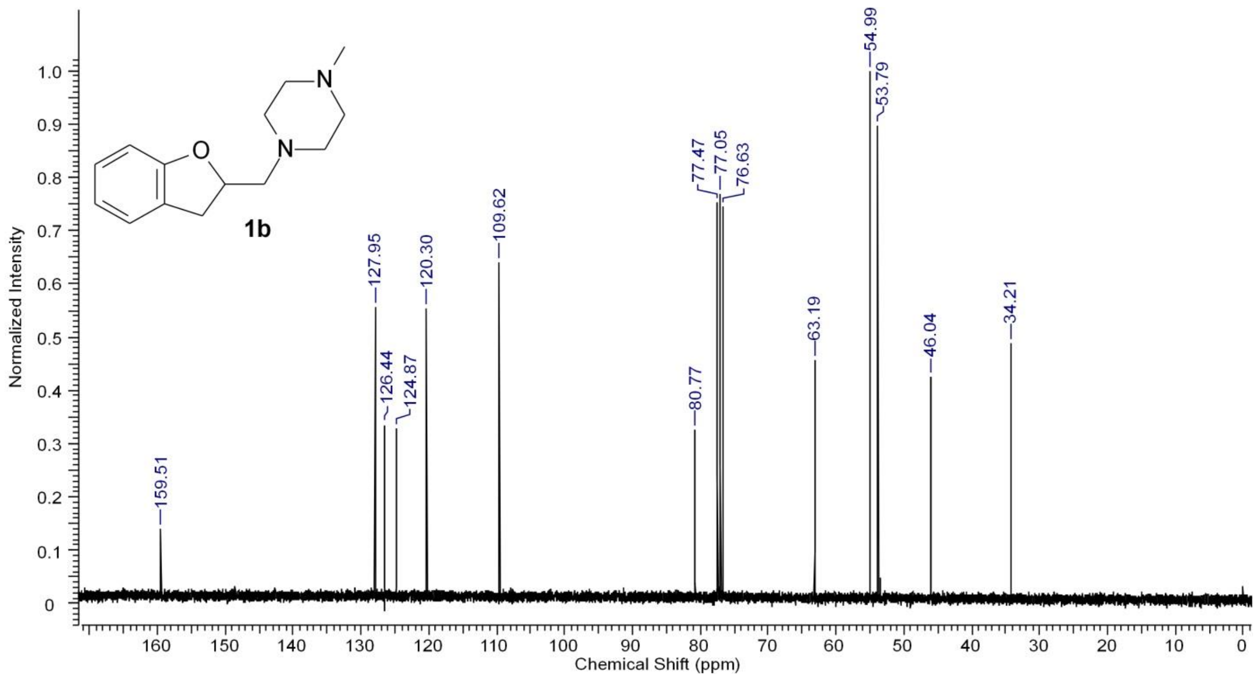

Supplementary Figure 6. Copy of the ^{13}C-NMR spectra of compound **1b**.

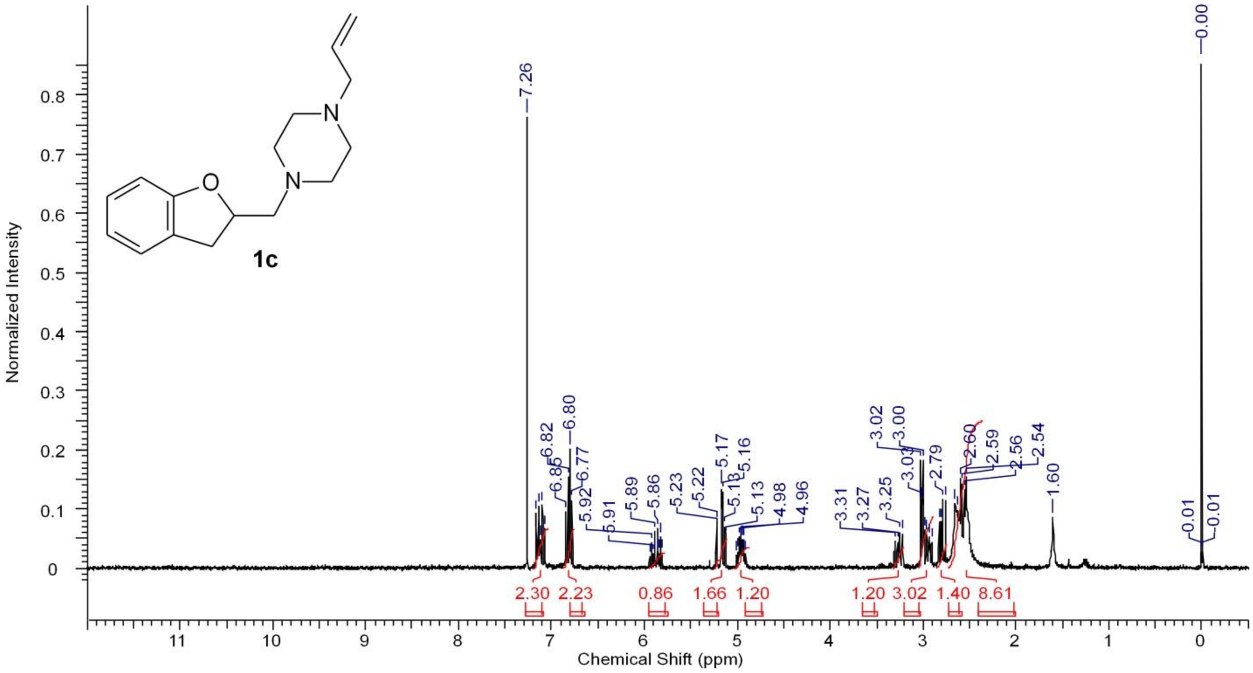

Supplementary Figure 7. Copy of the ^{1}H-NMR spectra of compound **1c**.

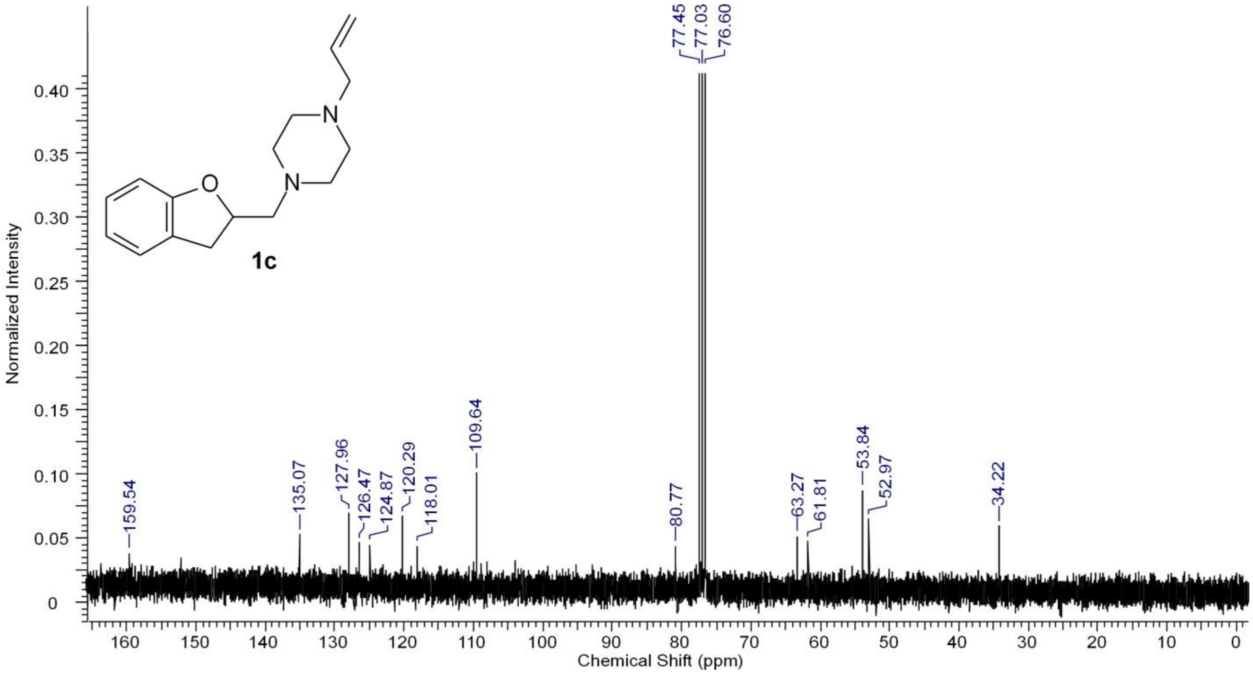

Supplementary Figure 8. Copy of the ^{13}C-NMR spectra of compound **1c**.

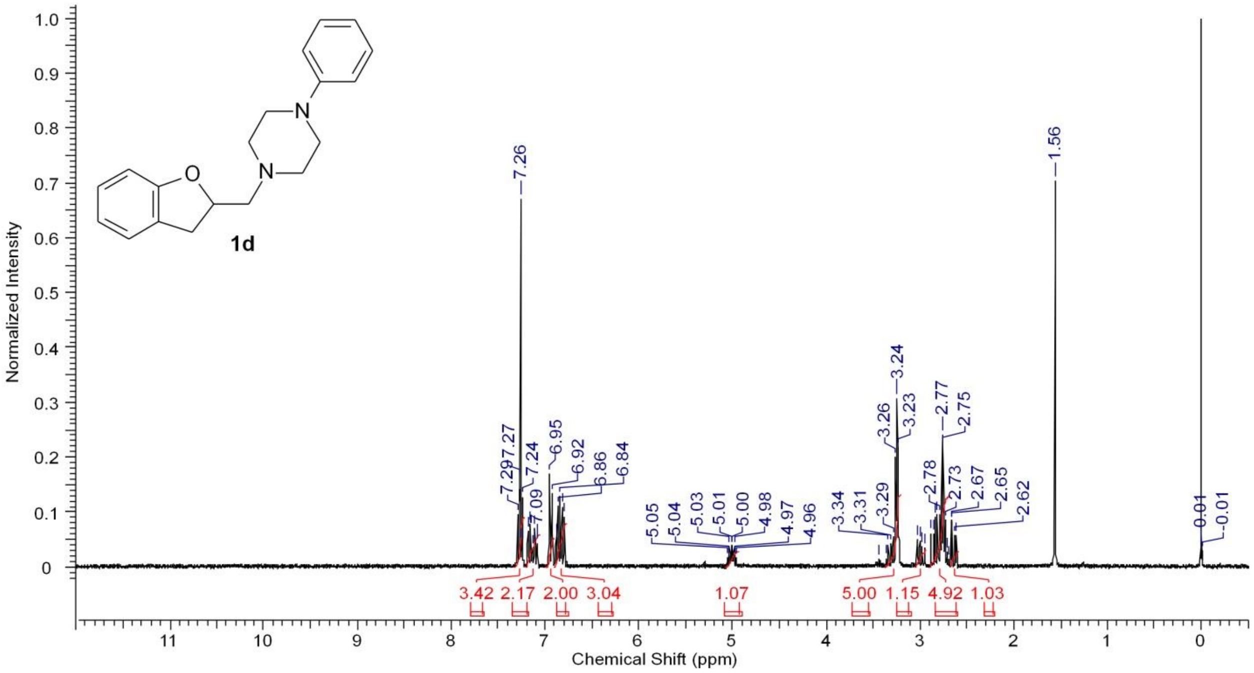

Supplementary Figure 9. Copy of the ^{1}H-NMR spectra of compound **1d**.

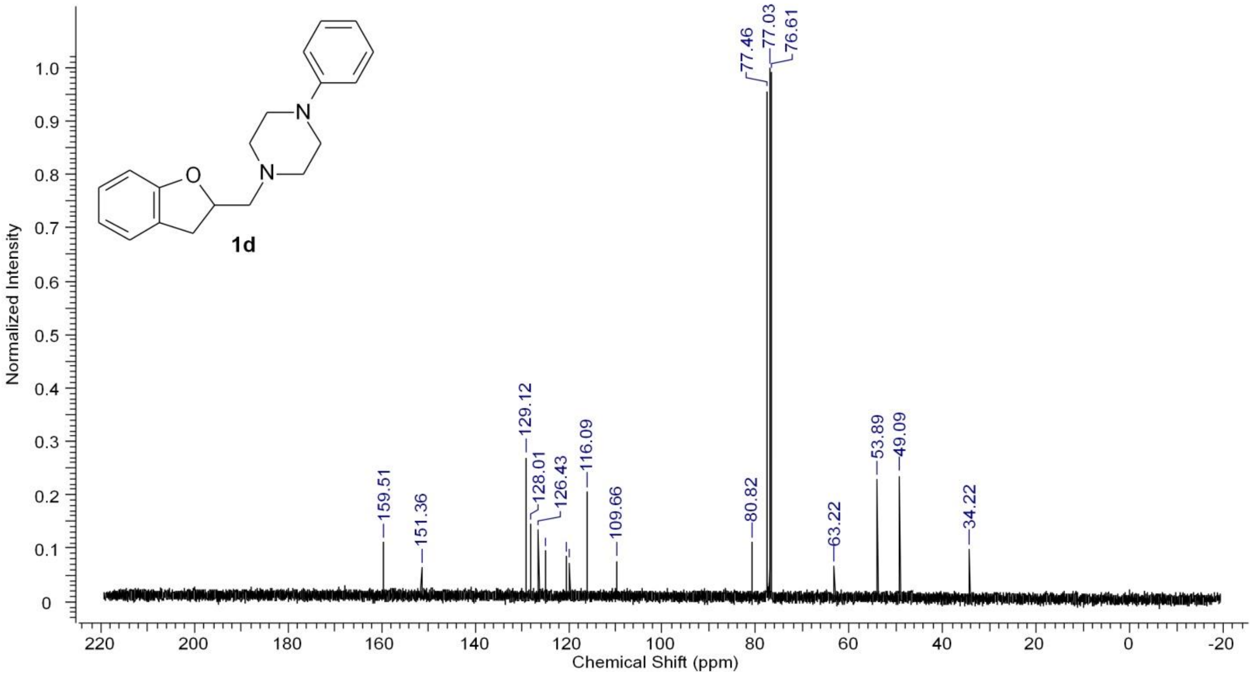

Supplementary Figure 10. Copy of the ^{13}C-NMR spectra of compound **1d**.

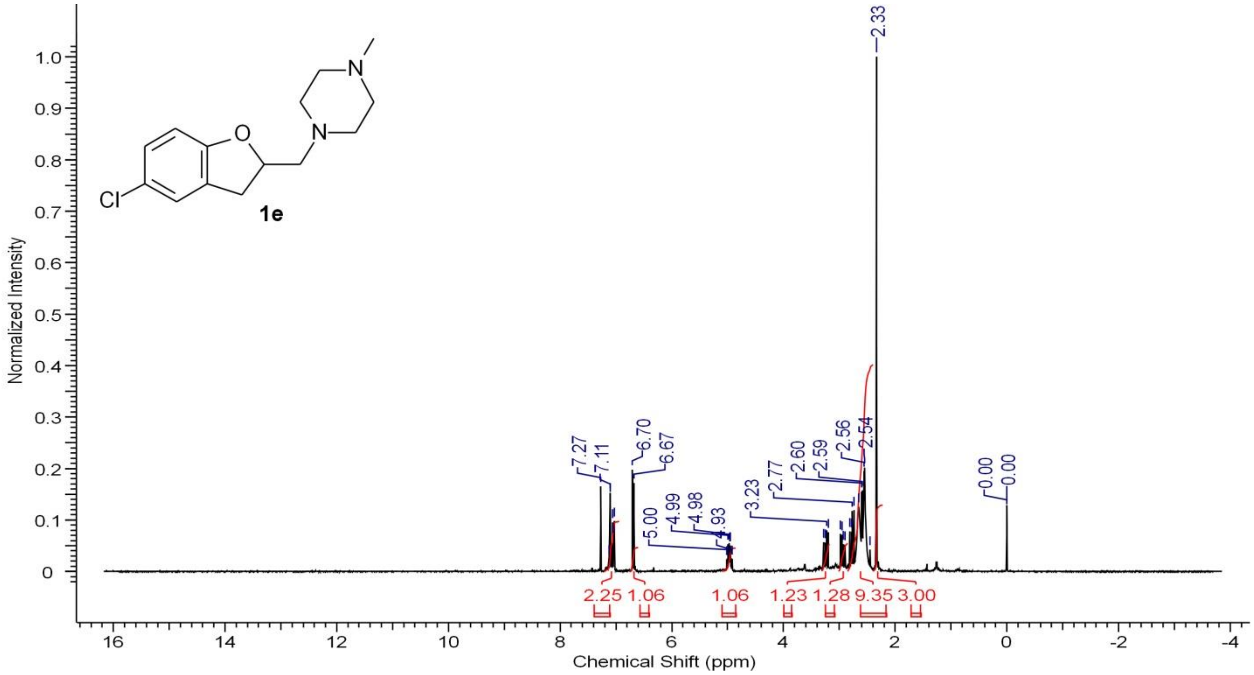

Supplementary Figure 11. Copy of the ^{1}H-NMR spectra of compound **1e**.

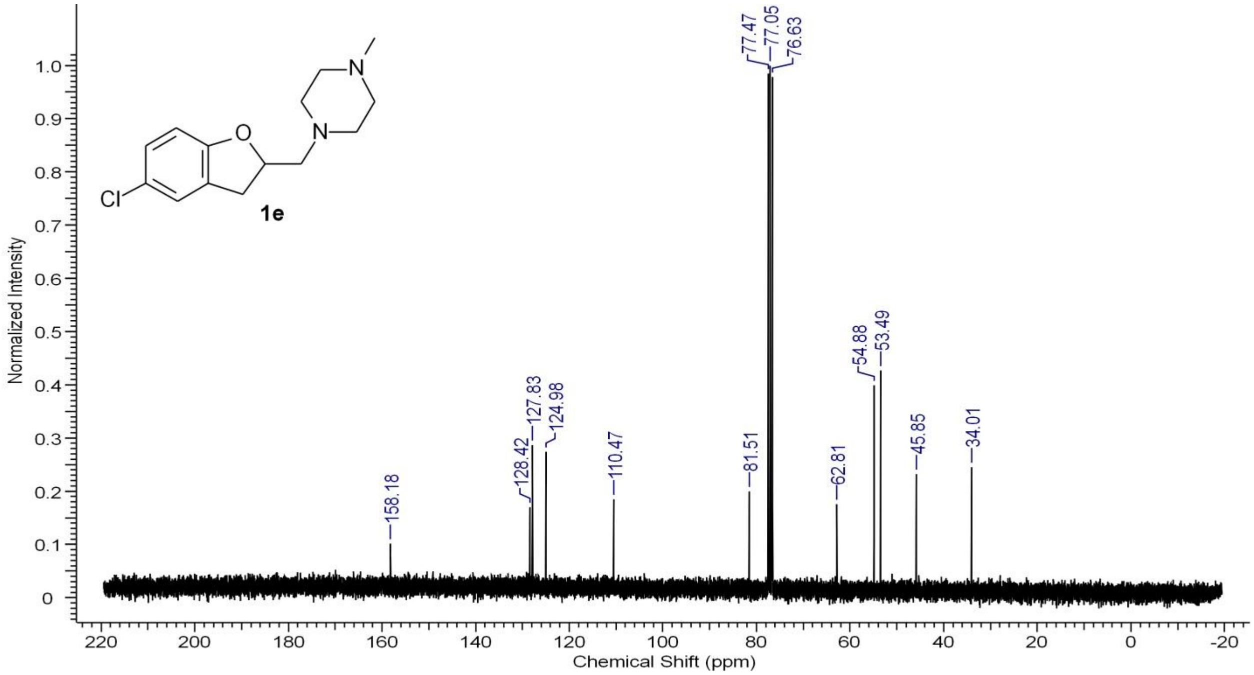

Supplementary Figure 12. Copy of the ^{13}C-NMR spectra of compound **1e**.

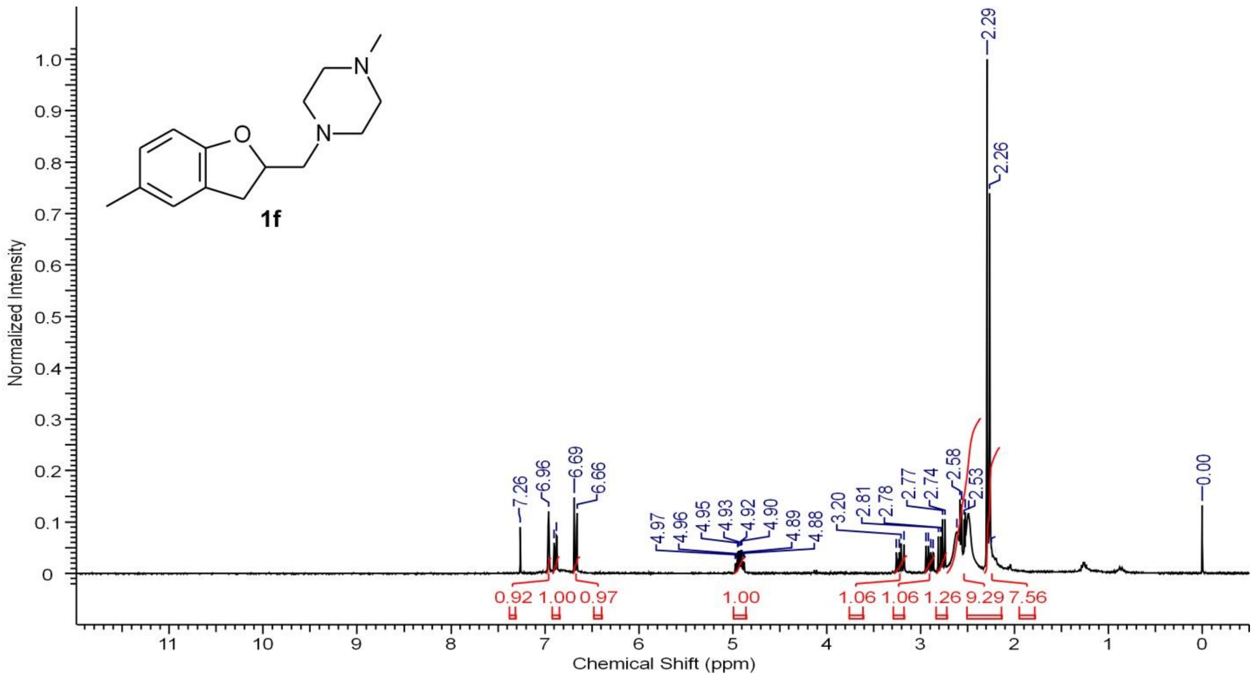

Supplementary Figure 13. Copy of the ^{1}H-NMR spectra of compound **1f**.

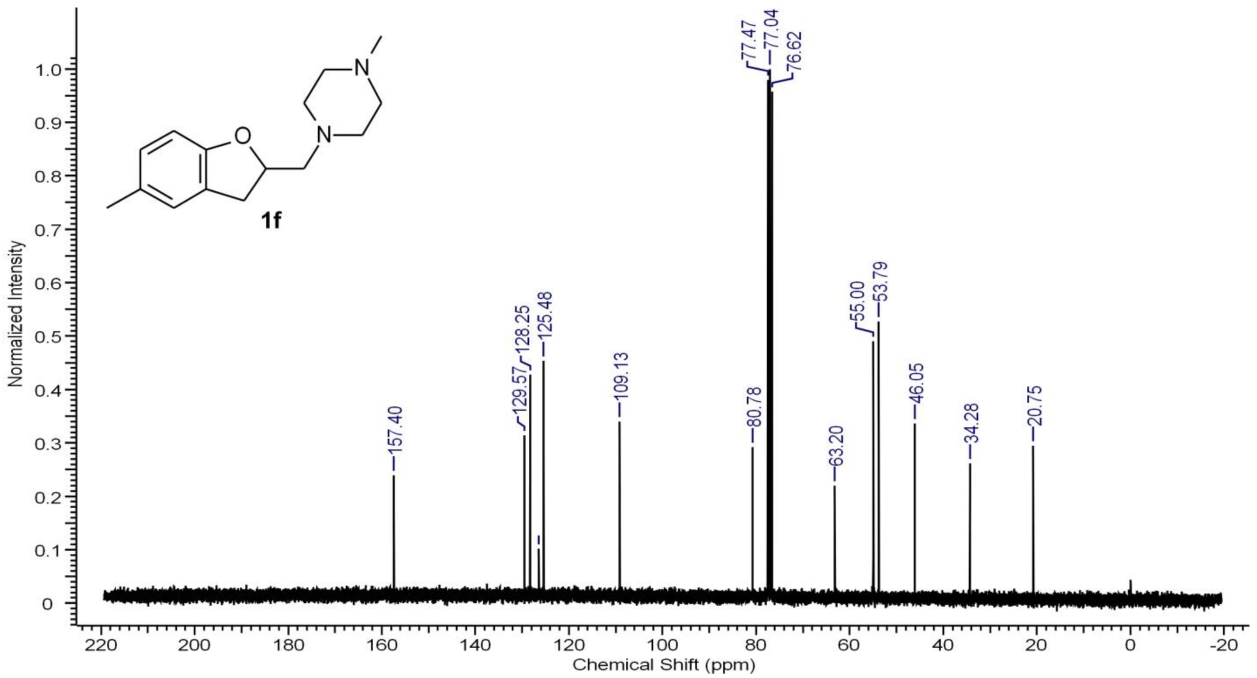

Supplementary Figure 14. Copy of the ^{13}C-NMR spectra of compound **1f**.

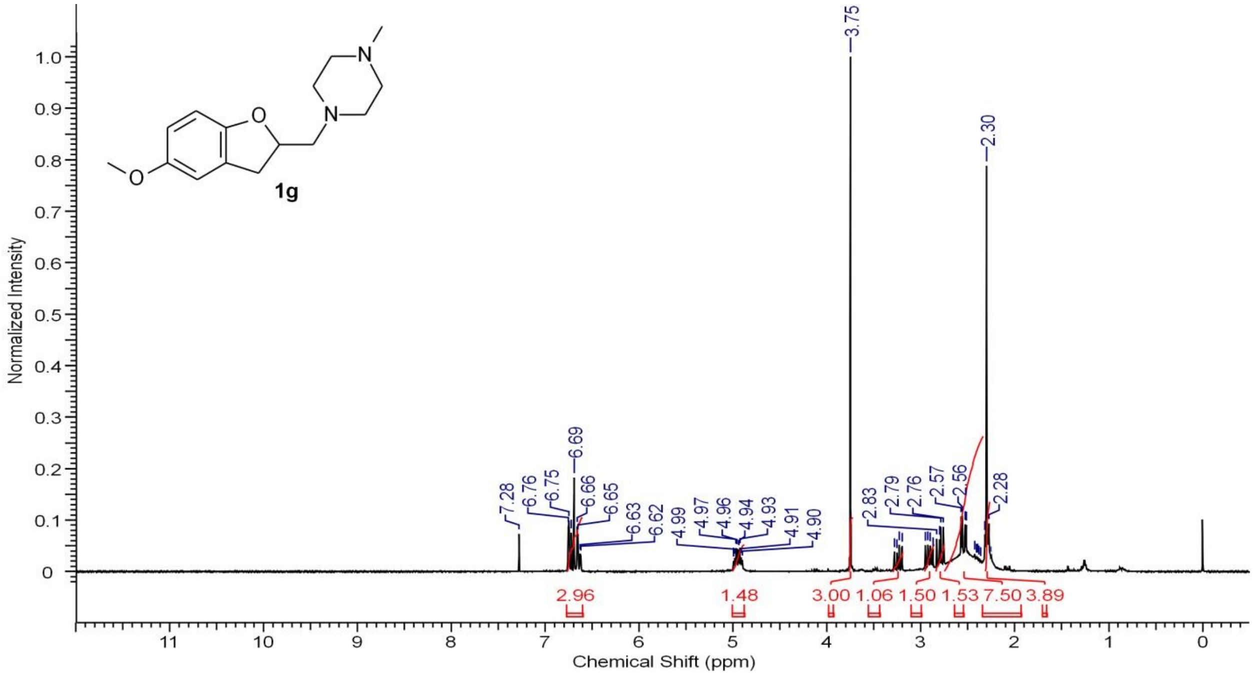

Supplementary Figure 15. Copy of the ^{1}H-NMR spectra of compound **1g**.

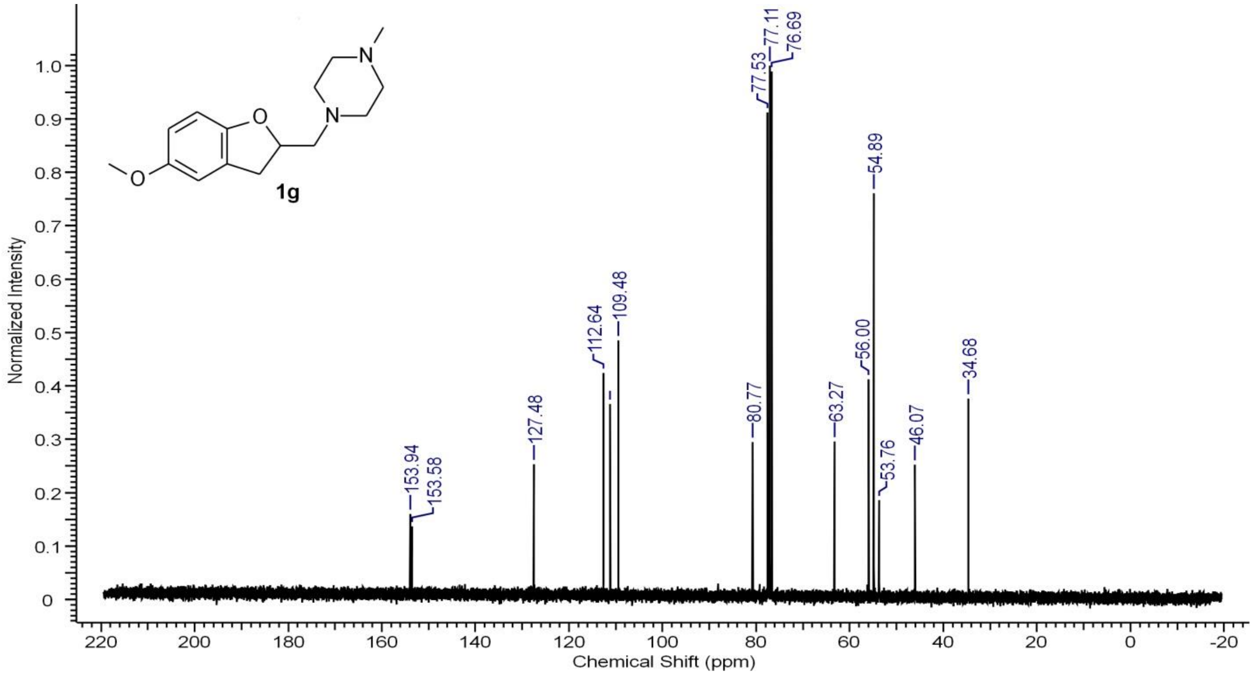

Supplementary Figure 16. Copy of the ^{13}C-NMR spectra of compound **1g**.

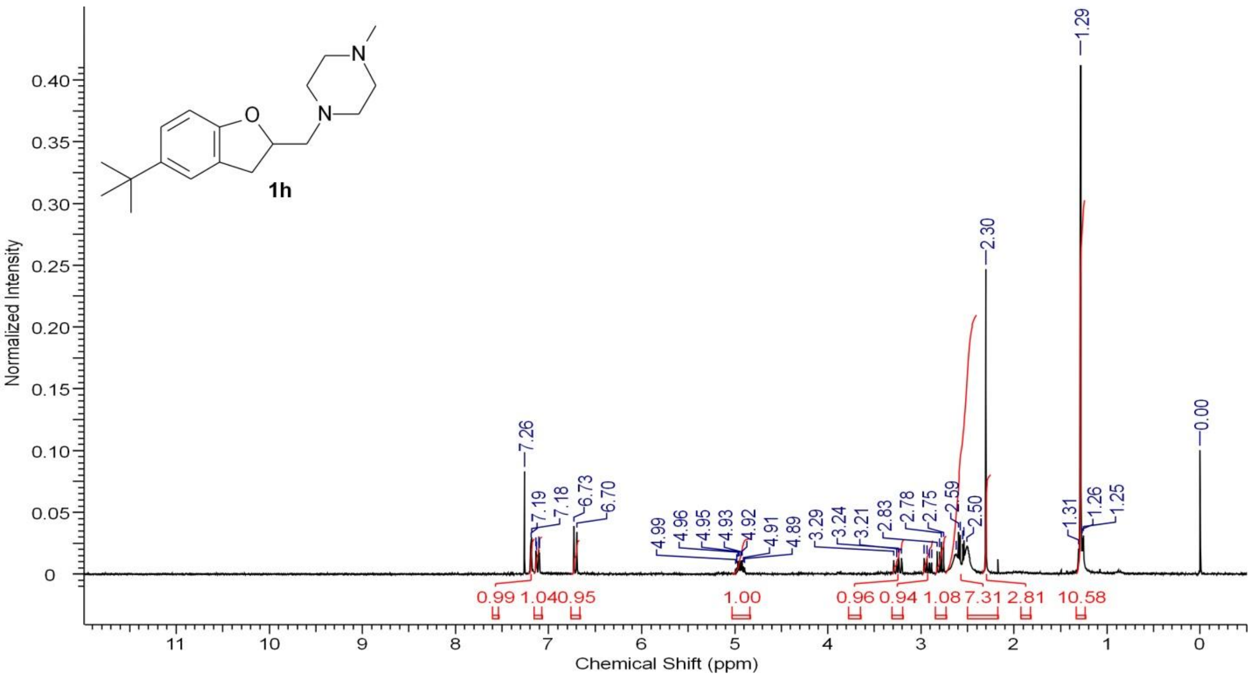

Supplementary Figure 17. Copy of the ^{1}H-NMR spectra of compound **1h**.

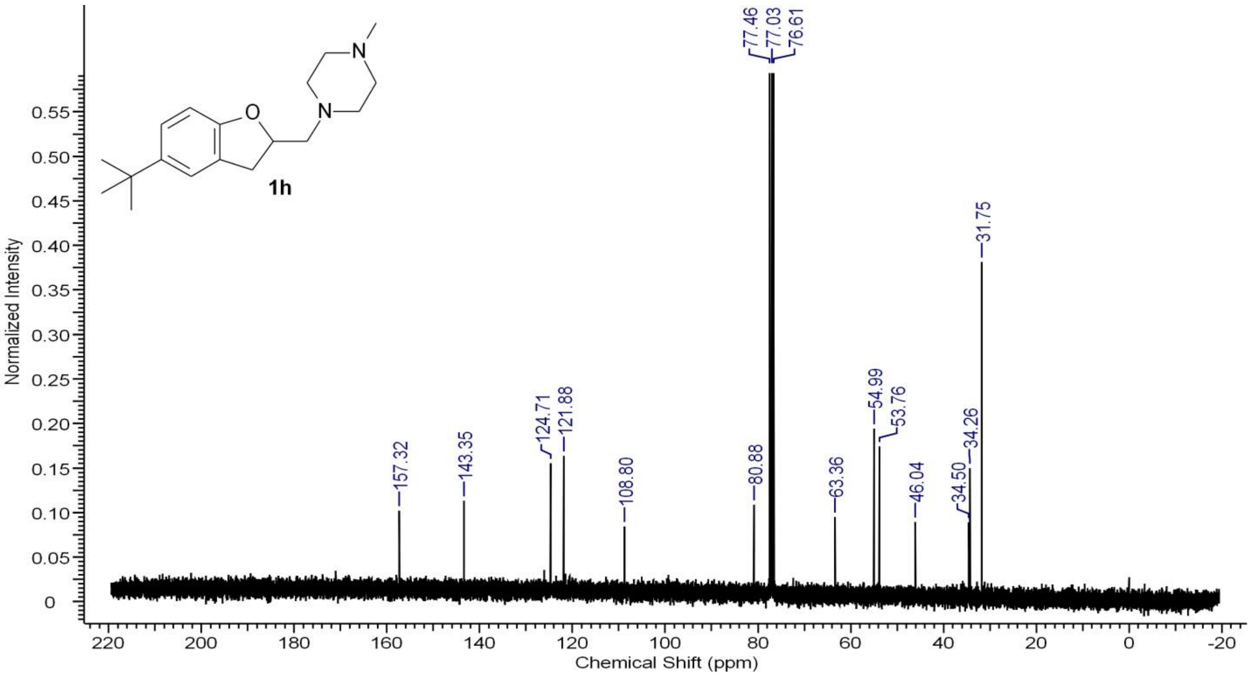

Supplementary Figure 18. Copy of the ^{13}C-NMR spectra of compound **1h**.

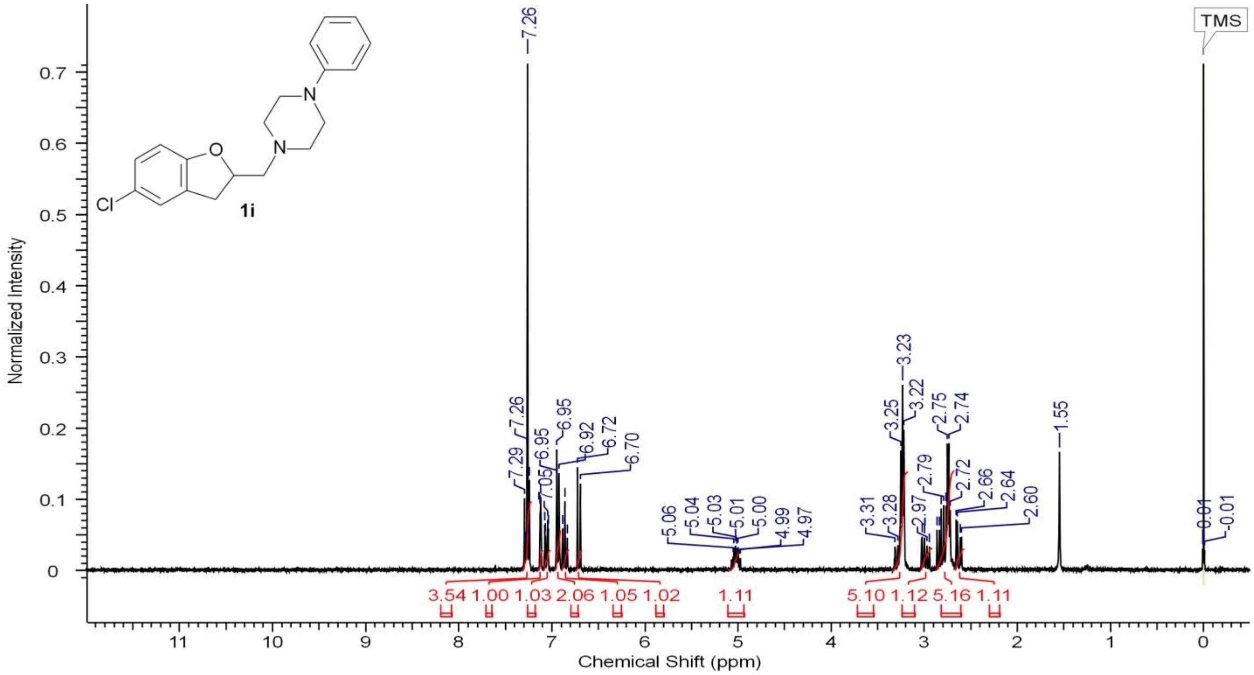

Supplementary Figure 19. Copy of the ^{1}H-NMR spectra of compound **1i**.

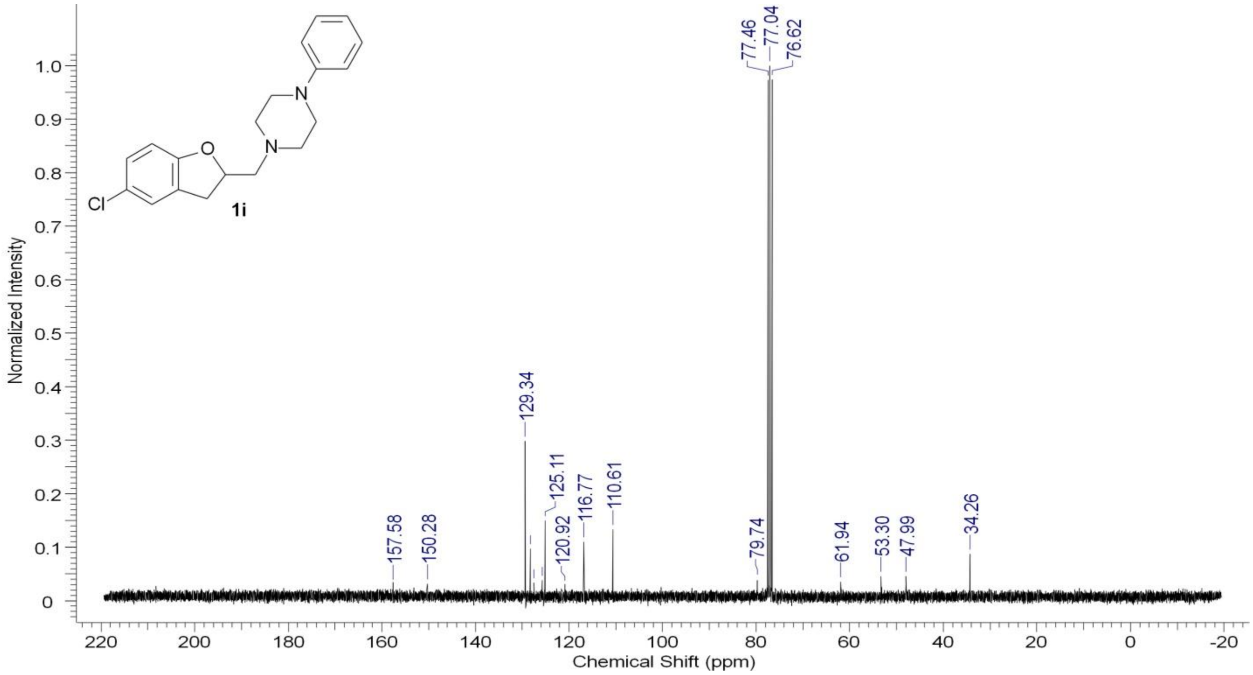

Supplementary Figure 20. Copy of the ^{13}C-NMR spectra of compound **1i**.
